# Supplementary material for: Structural details of a Class B GPCR-arrestin complex revealed by genetically encoded crosslinkers in living cells
Source: Nat Commun. 2023 Mar 1;14:1151. doi: 10.1038/s41467-023-36797-2 (PMC9977954; doi:10.1038/s41467-023-36797-2)
Supplement: Supplementary file 1 — Supplementary Information [file 41467_2023_36797_MOESM1_ESM.pdf]

Structural details of a Class B GPCR - arrestin complex revealed by genetically encoded crosslinkers in living cells

Authors

Yasmin Aydin[#1], Thore Böttke[#1], Jordy Homing Lam[#2], Stefan Ernicke[1], Anna Fortmann[1], Maik Tretbar[4], Barbara Zarzycka[5], Vsevolod V. Gurevich[6], Vsevolod Katritch*[2,3] and Irene Coin*[1]

[1] Faculty of Life Science, Institute of Biochemistry, Leipzig University, Bruederstr. 34, 04103 Leipzig, Germany.

[2] Department of Quantitative and Computational Biology, University of Southern California, Los Angeles, CA, USA.

[3] Department of Chemistry, Bridge Institute, USC Michelson Center for Convergent Biosciences, University of Southern California, Los Angeles, CA, USA.

[4] Medical Faculty, Institute for Drug Discovery, Leipzig University, Bruederstr. 34, 04103 Leipzig, Germany.

[5] Division of Medicinal Chemistry, Amsterdam Institute of Molecular and Life Sciences (AIMMS), Faculty of Science, Vrije Universiteit Amsterdam, De Boelelaan 1108, 1081 HZ Amsterdam, The Netherlands.

[6] Department of Pharmacology, Vanderbilt University, Nashville, TN 37232-0146, USA.

[#]These authors contributed equally to the work
*To whom correspondence shall be addressed: katritch@usc.edu; irene.coin@uni-leipzig.de

Supplementary Figures and Tables

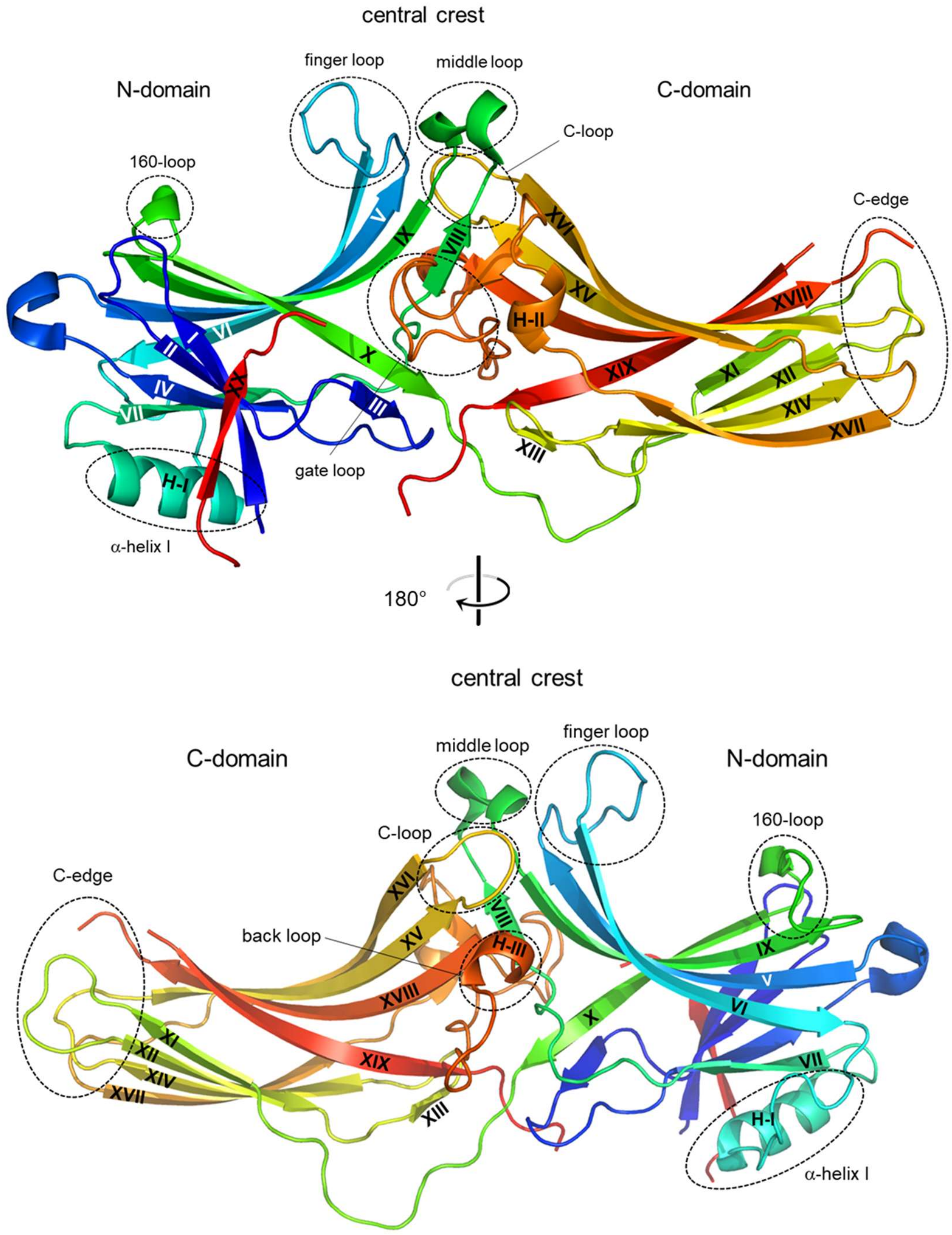

Supplementary Fig. 1: Structural overview of arr2 (PDBID: 1g4m[1]). N-terminus in blue, C-terminus in red. All β-strands and α-helices are consecutively numbered, following the chain. Regions mentioned in the text are indicated by dotted circles. The N-domain includes the α-helix I and the 160-loop. The gate loop and the C-edge are part of the C-domain. The "central crest" comprises three loops: the finger loop, the middle loop and the C-loop.

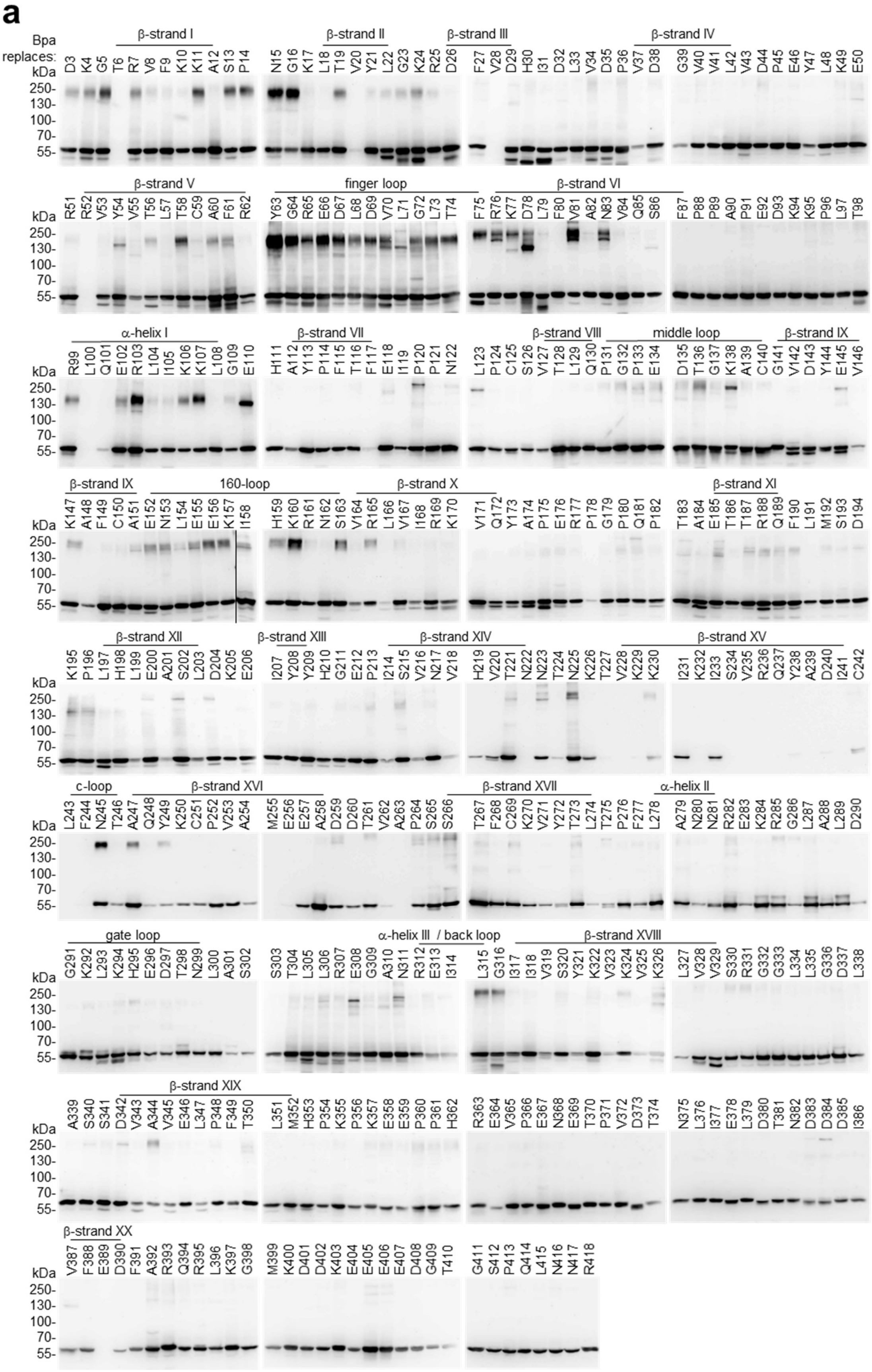
a
Bpa replaces:
kDa
250- 130- 100- 70- 55-
β-strand I
β-strand II
β-strand III
β-strand IV
D3 K4 G5 T6 R7 V8 F9 K10 K11 A12 S13 P14
N15 G16 K17 L18 T19 V20 Y21 L22 G23 K24 R25 D26
F27 V28 D29 H30 I31 D32 L33 V34 D35 P36 V37 D38
G39 V40 V41 L42 V43 D44 P45 E46 Y47 L48 K49 E50
β-strand V
finger loop
β-strand VI
R51 R52 V53 Y54 V55 T56 L57 T58 C59 A60 F61 R62
Y63 G64 R65 E66 D67 L68 D69 V70 L71 G72 L73 T74
F75 R76 K77 D78 L79 F80 V81 A82 N83 V84 Q85 S86
F87 P88 P89 A90 P91 E92 D93 K94 K95 P96 L97 T98
α-helix I
β-strand VII
β-strand VIII
middle loop
β-strand IX
R99 L100 Q101 E102 R103 L104 I105 K106 K107 L108 G109 E110
H111 A112 Y113 P114 F115 T116 F117 E118 I119 P120 P121 N122
L123 P124 C125 S126 V127 T128 L129 Q130 P131 G132 P133 E134
D135 T136 G137 K138 A139 C140 G141 V142 D143 Y144 E145 V146
β-strand IX
160-loop
β-strand X
β-strand XI
K147 A148 F149 C150 A151 E152 N153 L154 E155 E156 K157 I158
H159 K160 R161 N162 S163 V164 R165 L166 V167 I168 R169 K170
V171 Q172 Y173 A174 P175 E176 R177 P178 G179 P180 Q181 P182
T183 A184 E185 T186 T187 R188 Q189 F190 L191 M192 S193 D194
β-strand XII
β-strand XIII
β-strand XIV
β-strand XV
K195 P196 L197 H198 L199 E200 A201 S202 L203 D204 K205 E206
I207 Y208 Y209 H210 G211 E212 P213 I214 S215 V216 N217 V218
H219 V220 T221 N222 N223 T224 N225 K226 T227 V228 K229 K230
I231 K232 I233 S234 V235 R236 Q237 Y238 A239 D240 I241 C242
c-loop
β-strand XVI
β-strand XVII
α-helix II
L243 F244 N245 T246 A247 Q248 Y249 K250 C251 P252 V253 A254
M255 E256 E257 A258 D259 D260 T261 V262 A263 P264 S265 S266
T267 F268 C269 K270 V271 Y272 T273 L274 T275 P276 F277 L278
A279 N280 N281 R282 E283 K284 R285 G286 L287 A288 L289 D290
gate loop
α-helix III / back loop
β-strand XVIII
G291 K292 L293 K294 H295 E296 D297 T298 N299 L300 A301 S302
S303 T304 L305 L306 R307 E308 G309 A310 N311 R312 E313 I314
L315 G316 I317 I318 V319 S320 Y321 K322 V323 K324 V325 K326
L327 V328 V329 S330 R331 G332 G333 L334 L335 G336 D337 L338
β-strand XIX
A339 S340 S341 D342 V343 A344 V345 E346 L347 P348 F349 T350
L351 M352 H353 P354 K355 P356 K357 E358 E359 P360 P361 H362
R363 E364 V365 P366 E367 N368 E369 T370 P371 V372 D373 T374
N375 L376 I377 E378 L379 D380 T381 N382 S383 D384 D385 I386
β-strand XX
V387 F388 E389 D390 F391 A392 R393 Q394 R395 L396 K397 G398
M399 K400 D401 D402 K403 E404 E405 E406 E407 D408 G409 T410
G411 S412 P413 Q414 L415 N416 N417 R418

b

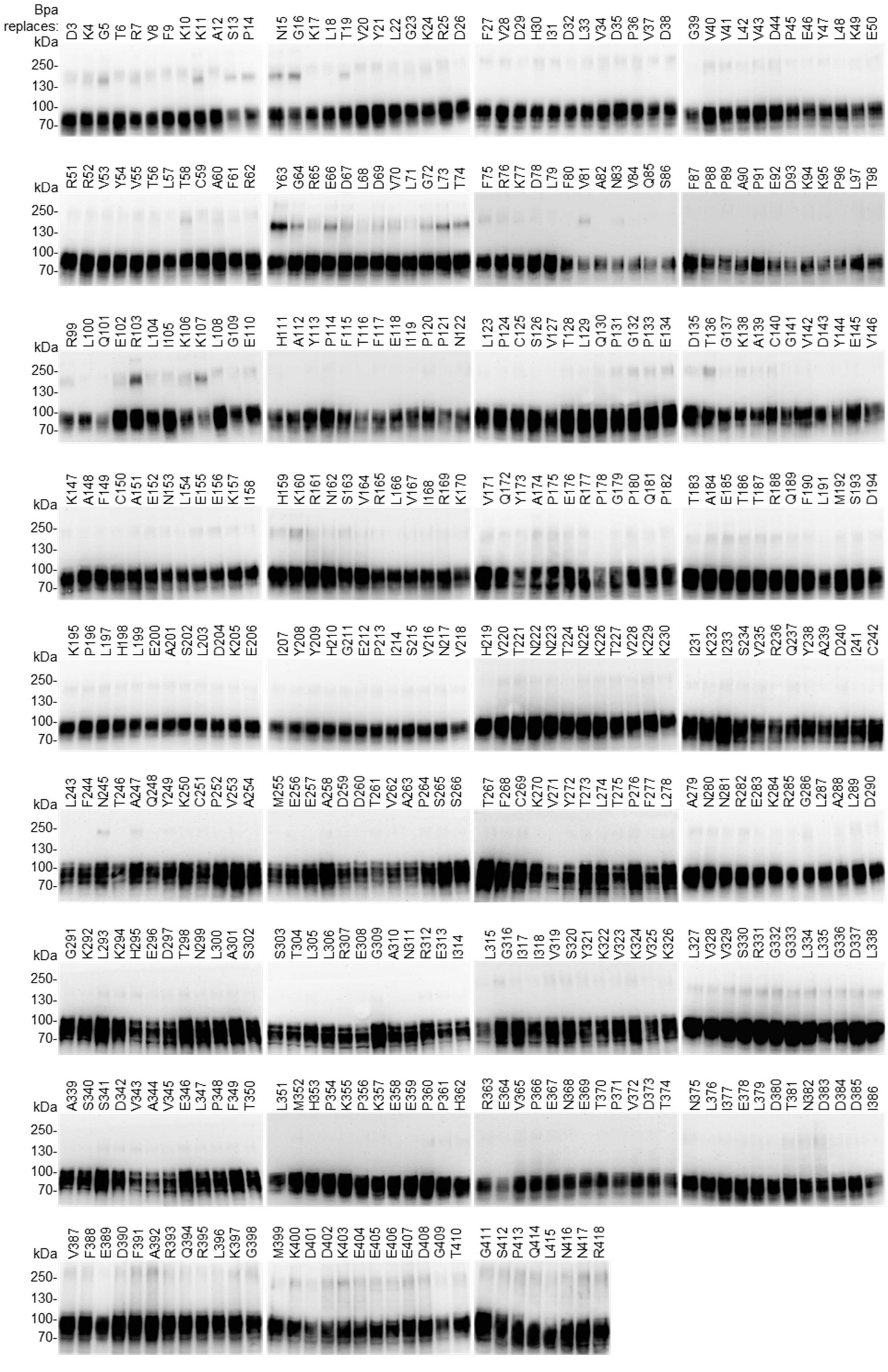

Supplementary Fig. 2: Photocrosslinking map of the arr2-PTH1R complex. Indicated arr2 positions were exchanged for Bpa and combined with the PTH1R. After agonist stimulation, cells were irradiated with UV-light (365 nm). Whole cell lysates (n=1) were resolved on Tris-glycine gels and analyzed by immunostaining. Arr2 was detected by an α-HA antibody (a), the PTH1R (b) using an α-PTH1R antibody. Arrestin runs at approximately ~55 kDa, PTH1R runs at an apparent molecular weight of ~70-100 kDa, the crosslinked PTH1R-arr2-complex at ~200 kDa. Uncropped blots are provided as a Source Data file.

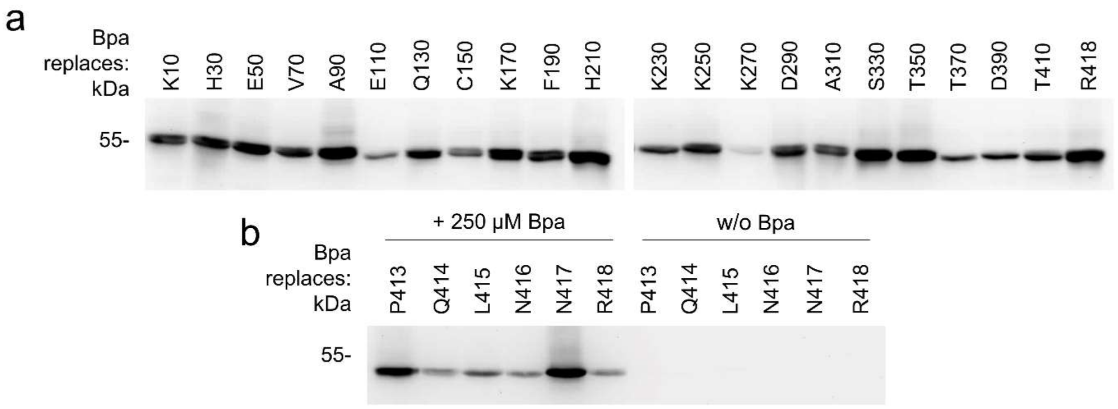

Supplementary Fig. 3: Expression of Bpa-containing arr2 mutants. (a) Western blot analysis of cell lysates (n=1); samples from the same experiment were run in parallel. Representative positions in arrestin for stop codon suppression were chosen every 20 residues. The yields of Bpa incorporation remained comparable throughout the protein. (b) Western blot analysis of cell lysates (n=1) to rule out a read through of the amber stop codon in the last six C-terminal positions of arr2. We compared the expression levels of the detected product in the presence and absence of Bpa. Arr2 could only be detected with its C-terminal 3xHA-tag if 250 µM Bpa were present in the cell culture media. Uncropped blots are provided as a Source Data file.

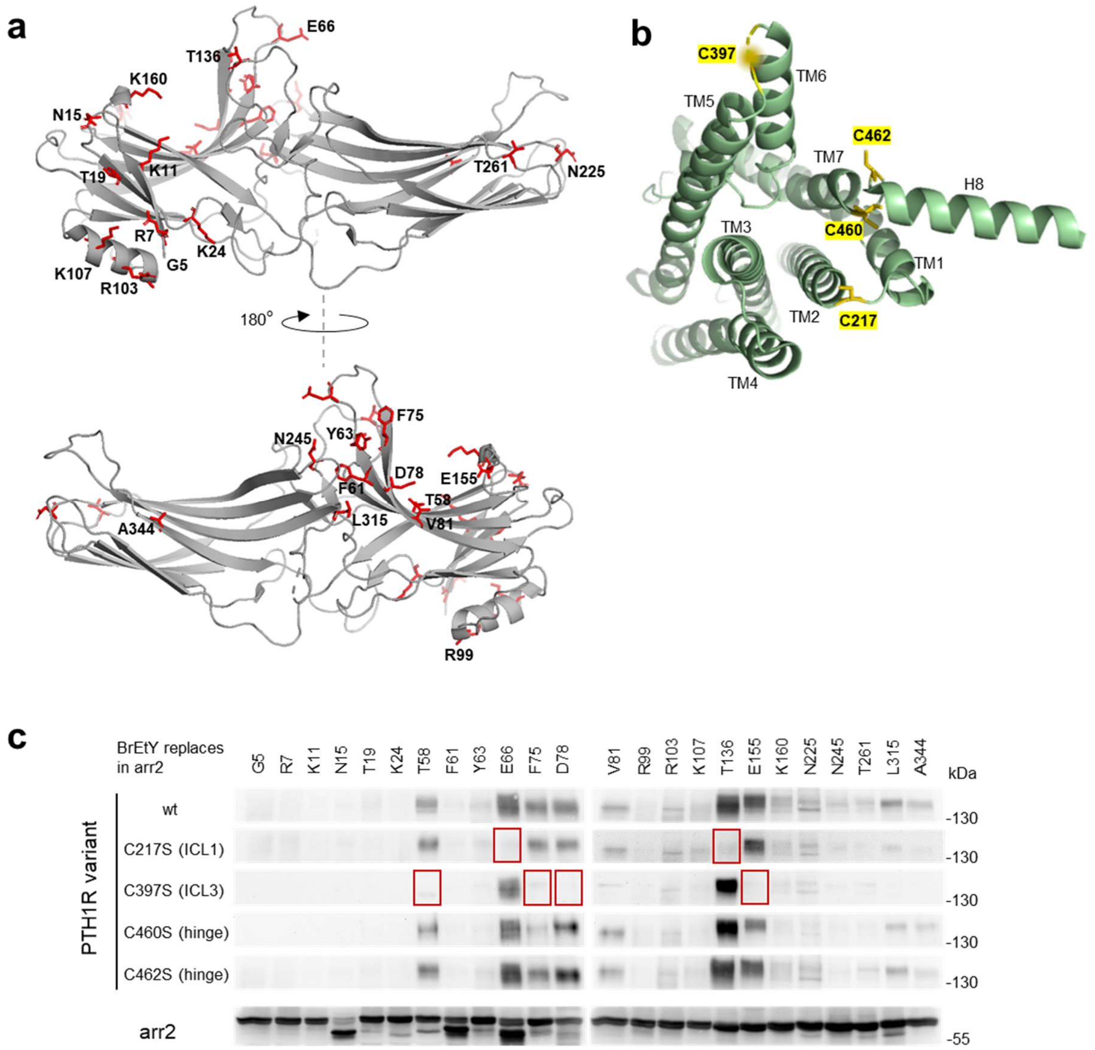

Supplementary Fig. 4: Chemical crosslinking, unbiased search. (a) Cartoon representation of receptor-bound rat arr2 (PDBID: 4jqi[2]). 24 photo-crosslinking hits sampling the whole footprint of PTH1R were chosen for pairwise chemical crosslinking (shown as red stick models). (b) Cartoon representation of active PTH1R as seen from the intracellular side (PDBID: 6nbf[3]). Naturally occurring Cys residues are shown in yellow. C217 is in ICL1, C397 in ICL3, C460 and C462 are in the hinge between helix VII and helix VIII. (c) Western blot of cell lysates (n=1) stained with an α-HA antibody. Residues of arrestin replaced with BrEtY are indicated in the upper row, mutations at the PTH1R are indicated on the left side. A crosslinking signal that vanishes upon Cys to Ser substitution (red boxes) reveals which PTH1R Cys was captured by the corresponding BrEtY-arrestin. Uncropped blots are provided as a Source Data file.

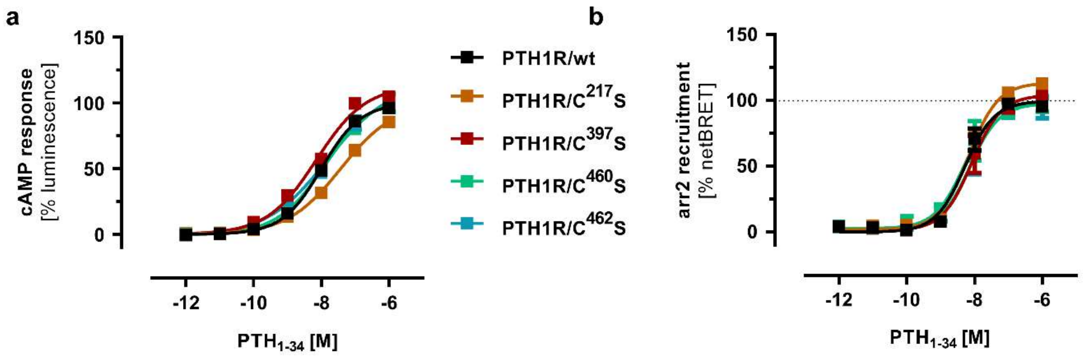

	wt	$C^{217}S$	$C^{397}S$	$C^{460}S$	$C^{462}S$
**EC_{50} [nM]**	**10**	**40**	**5**	**9**	**15**
95 % CI	3 – 29	23 – 70	1 – 30	3 – 26	7 – 35

	wt	$C^{217}S$	$C^{397}S$	$C^{460}S$	$C^{462}S$
**$BRET_{50}$ [nM]**	**5**	**7**	**8**	**5**	**7**
95 % CI	4 – 8	5 – 9	5 – 14	3 – 9	4 – 14

Supplementary Fig. 5: Activation and arrestin recruitment at Ser-PTH_1R variants. (a) Activation of PTH1R and mutants by PTH(1-34) (G_s activation). The assay measures cAMP accumulation in live HEK293T cells stimulated with indicated concentrations of PTH(1-34) based on CRE-controlled expression of a luciferase reporter. Raw luminescence data of the reporter luciferase were normalized to the transfection control and set to 100% activation for each receptor variant. Plot shows means ± SEM of n=1 representative experiment. The table shows the geometric mean of the EC_{50} values of n=4 independent experiments with the 95% confidence interval (CI). (b) Concentration-response curves for recruitment of Venus-arr2 to PTH_1R-Nluc as measured by BRET. Plot shows means ± SEM of n=3 independent experiments. The table shows the geometric mean of the $BRET_{50}$ values from n=3 independent experiments with 95% confidence intervals. Source data are provided as a Source Data file.

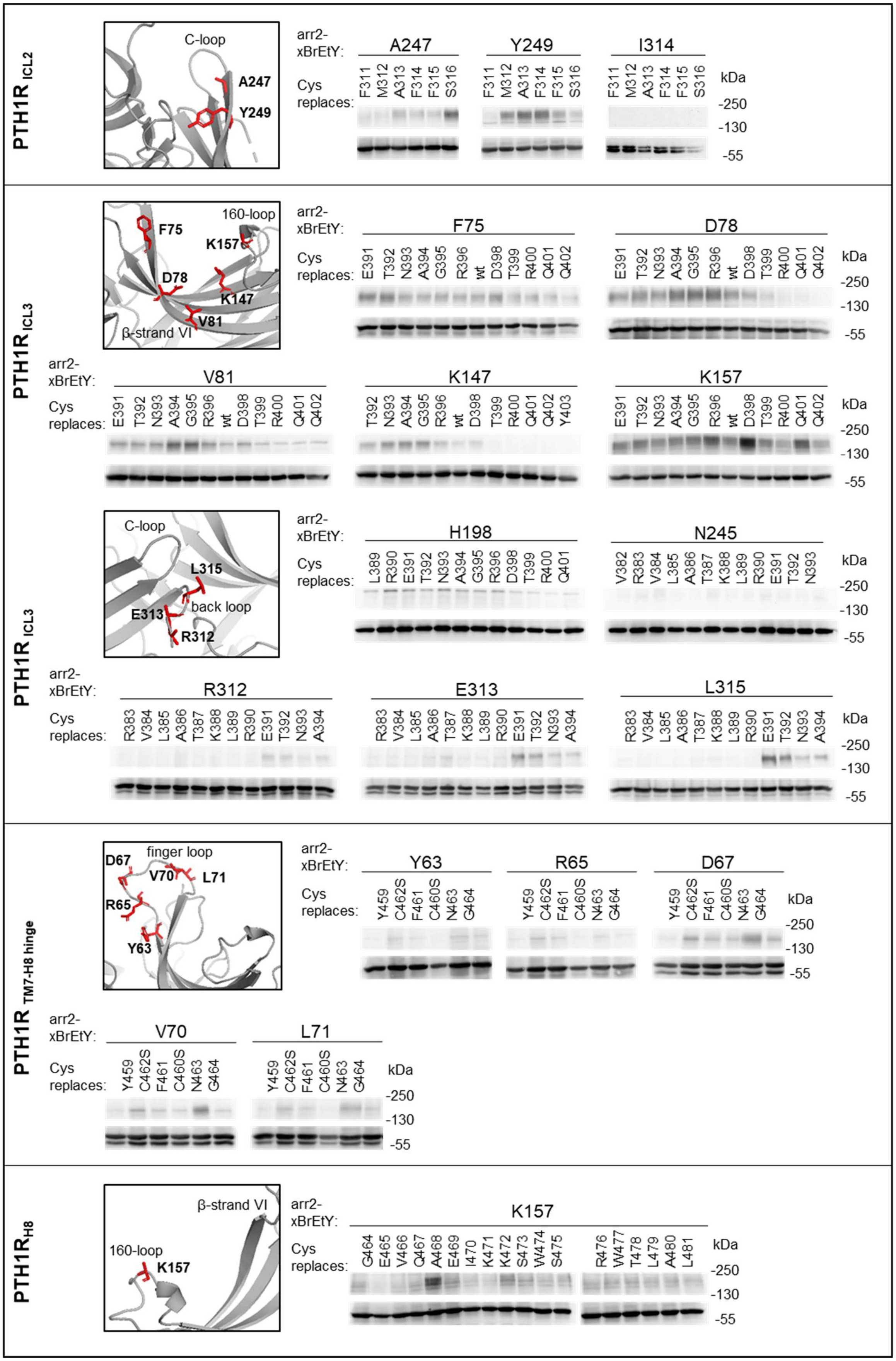

PTH1R ICL2
C-loop
A247
Y249
arr2-xBrEtY:
A247
Y249
I314
Cys replaces:
F311 M312 A313 F314 F315 S316
kDa
-250
-130
-55
PTH1R ICL3
F75
160-loop
K157
D78
K147
V81
β-strand VI
F75
D78
E391 T392 N393 A394 G395 R396 wt D398 T399 R400 Q401 Q402
V81
K147
T392 N393 A394 G395 R396 wt D398 T399 R400 Q401 Q402 Y403
K157
PTH1R ICL3
C-loop
L315
back loop
E313
R312
H198
L389 R390 E391 T392 N393 A394 G395 R396 D398 T399 R400 Q401
N245
V382 R383 V384 L385 A386 T387 K388 L389 R390 E391 T392 N393
R312
E313
L315
R383 V384 L385 A386 T387 K388 L389 R390 E391 T392 N393 A394
PTH1R TM7-H8 hinge
D67
finger loop
V70
L71
R65
Y63
Y63
R65
D67
V70
L71
Y459 C462S F461 C460S N463 G464
PTH1R H8
β-strand VI
160-loop
K157
K157
G464 E465 V466 Q467 A468 E469 I470 K471 K472 S473 W474 S475
R476 W477 T478 L479 A480 L481

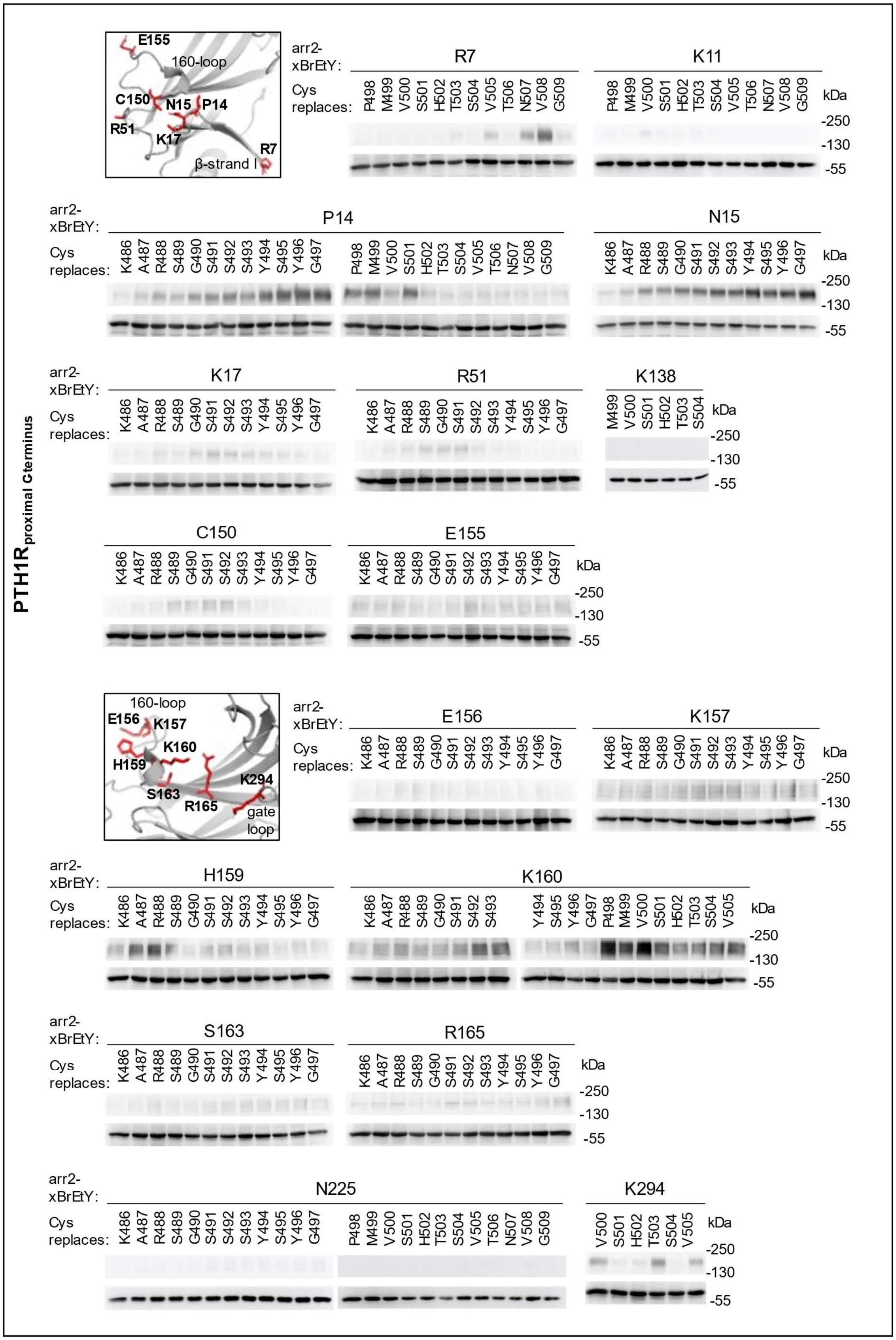
PTH1R proximal Cterminus
E155
160-loop
C150
N15
P14
R51
K17
R7
β-strand I
arr2-xBrEtY:
Cys replaces:
R7
P498 M499 V500 S501 H502 T503 S504 V505 T506 N507 V508 G509
K11
P498 M499 V500 S501 H502 T503 S504 V505 T506 N507 V508 G509
kDa
-250
-130
-55
P14
K486 A487 R488 S489 G490 S491 S492 S493 Y494 S495 Y496 G497
P498 M499 V500 S501 H502 T503 S504 V505 T506 N507 V508 G509
N15
K486 A487 R488 S489 G490 S491 S492 S493 Y494 S495 Y496 G497
K17
K486 A487 R488 S489 G490 S491 S492 S493 Y494 S495 Y496 G497
R51
K486 A487 R488 S489 G490 S491 S492 S493 Y494 S495 Y496 G497
K138
M499 V500 S501 H502 T503 S504
C150
K486 A487 R488 S489 G490 S491 S492 S493 Y494 S495 Y496 G497
E155
K486 A487 R488 S489 G490 S491 S492 S493 Y494 S495 Y496 G497
160-loop
E156
K157
K160
H159
K294
S163
R165
gate loop
E156
K486 A487 R488 S489 G490 S491 S492 S493 Y494 S495 Y496 G497
K157
K486 A487 R488 S489 G490 S491 S492 S493 Y494 S495 Y496 G497
H159
K486 A487 R488 S489 G490 S491 S492 S493 Y494 S495 Y496 G497
K160
K486 A487 R488 S489 G490 S491 S492 S493
Y494 S495 Y496 G497 P498 M499 V500 S501 H502 T503 S504 V505
S163
K486 A487 R488 S489 G490 S491 S492 S493 Y494 S495 Y496 G497
R165
K486 A487 R488 S489 G490 S491 S492 S493 Y494 S495 Y496 G497
N225
K486 A487 R488 S489 G490 S491 S492 S493 Y494 S495 Y496 G497
P498 M499 V500 S501 H502 T503 S504 V505 T506 N507 V508 G509
K294
V500 S501 H502 T503 S504 V505

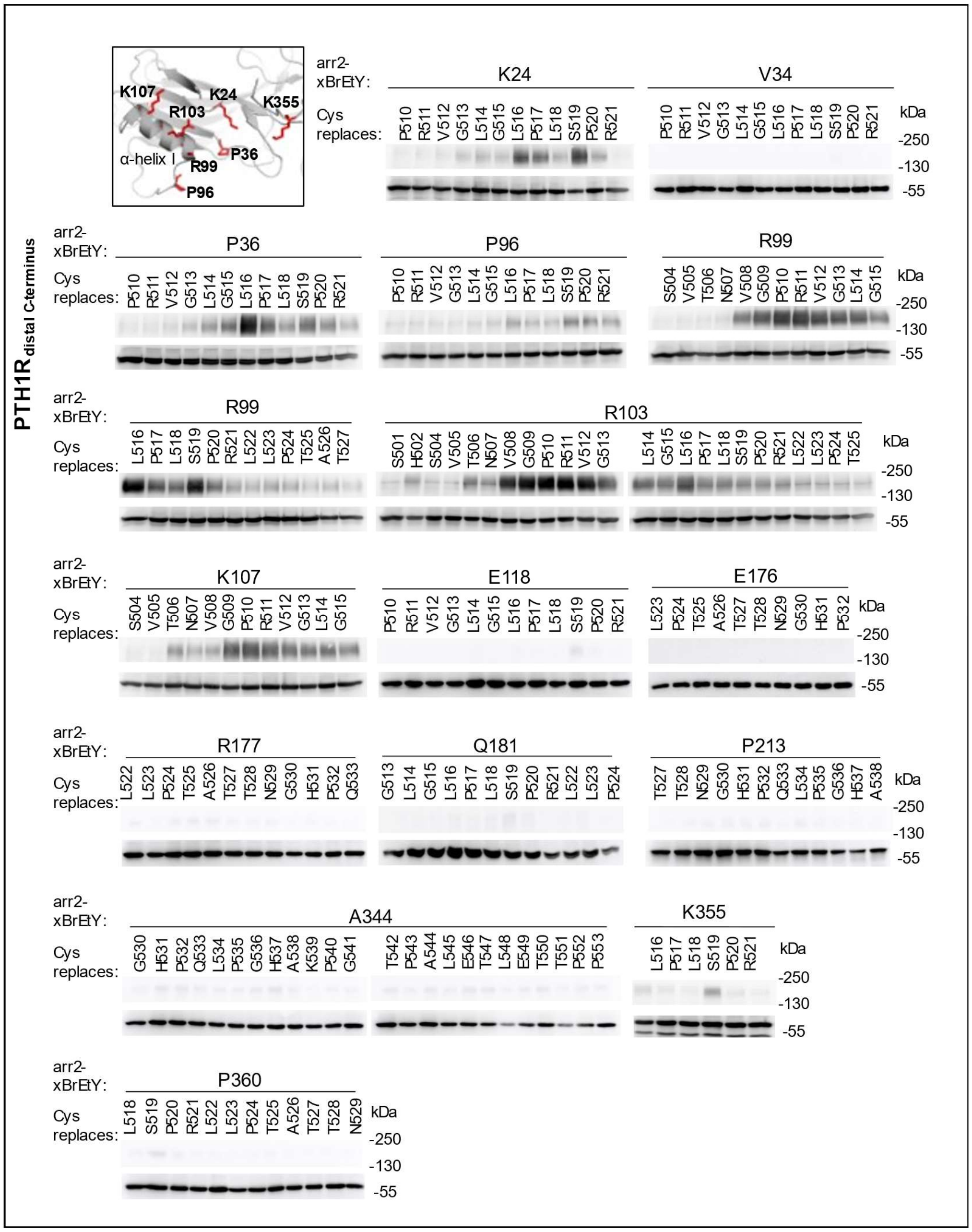

Supplementary Fig. 6: Chemical crosslinking, informed search. Representative western blots of cell lysates stained with an α-HA antibody. Residues of arr2 exchanged with BrEtY are indicated above groups of lanes, Cys mutations in the PTH1R are indicated on top of each lane. For each PTH1R region, BrEtY-arrestin positions giving crosslinking are visualized as red sticks in the active arr2 structure (PDBID: 4jqi). All tested combinations are shown. Experiments with detectable crosslinking were performed three times; signals were quantified by densitometry and used for the statistical analysis. Uncropped blots are provided as a Source Data file.

a

	arr2 / PTH1R	Y63	R65	E66	D67	V70	L71	F75	D78	V81	T136	K147	E155	K157	H198	N245	A247	Y249	R312	E313	I314	L315
ICL1	C217			25							60											
ICL2	F311																23	15				
	M312																23	44				
	A313																44	57				
	F314																37	54				
	F315																30	37				
	S316																46	31				
ICL3	V382																					
	R383																		1	6		3
	V384																		2	9		4
	L385																		2	7		3
	A386																		3	2		4
	T387																		2	8		10
	K388																		3	8		10
	L389																		3	8		6
	R390																		2	6		3
	E391							43	46	37				51					12	38		58
	T392							46	54	37		21		60					12	27		41
	N393							35	52	41		38		55					6	18		24
	A394							31	63	48		35		41					7	19		30
	G395							37	67	48		31		51								
	R396							30	67	30		25		67								
	C397							36	55	22		16	42	68								
	D398							49	57	33		15		69								
	T399							28	25	17		6		55								
	R400							36	24	14		4		52								
	Q401							28	16	16		3		66								
	Q402							23	7	16		3		39								
	Y403											3										
TM7 - hinge - H8	Y459	3	8		13	9	7															
	C460	13	19		24	16	17															
	F461	6	9		15	15	13															
	C462	6	7		15	23	10															
	N463	11	12		36	40	31															
	G464	9	8		15	13	14							19								
	E465													13								
	V466													18								
	Q467													21								
	A468													48								
	E469													23								
	I470													21								
	K471													18								
	K472													41								
	S473													27								
	W474													21								
	S475													26								
	R476													10								
	W477													16								
	T478													15								
	L479													20								
	A480													16								
	L481													12								

b

PTH1R \ arr2	R7	K11	P14	N15	K17	R51	Y63	R65	D67	V70	L71	P96	R99	R103	K107	K138	C150	E155	E156	K157	H159	K160	S163	R165	N225	K294
K486			12	32	5	3											2	8	3	27	34	18	5	9		
A487			26	**33**	9	4											6	7	12	**38**	**55**	26	7	13		
R488			**47**	**50**	21	13											9	18	13	**47**	**58**	33	15	17		
S489			**42**	43	22	20											17	16	15	**40**	**47**	29	15	16		
G490			49	52	31	20											18	14	15	**39**	44	28	16	19		
S491			**55**	54	**37**	22											24	15	11	32	45	**35**	16	22		
S492			**51**	54	28	11											25	17	5	**44**	**44**	**40**	**25**	18		
S493			**51**	**57**	27	6											20	15	6	**44**	**32**	**36**	24	17		
Y494			**67**	**73**	26	8											17	10	6	**46**	34	**35**	25	19		
S495			**69**	67	20	4											10	16	4	**42**	30	**39**	19	22		
Y496			**63**	**67**	17	3											6	18	6	**46**	32	**53**	20	25		
G497			**66**	**77**	11	3											3	16	7	34	23	**53**	18	28		
P498	2		50																			**65**				
M499	4		51																			**70**				
V500	3		33																			**69**				28
S501	3		47																			**74**				11
H502	4		27											12								38				13
T503	10		24											44								**51**				**43**
S504	4		20										7	18	5							29				7
V505	20		23										19	14	18							30				28
T506	7		20										25	**51**	50											
N507	28		15										16	**35**	29											
V508	42		19										59	**63**	38											
G509	6		14									10	70	60	**71**											

c

C-terminus	PTH1R \ arr2	K24	V34	P36	P96	R99	R103	K107	E118	E176	R177	Q181	P213	A344	K355	P360
	P510	7		5	14	**77**	**65**	**76**								
	R511	17		9	16	**73**	**69**	**68**								
	V512	15		12	8	**65**	**68**	**65**								
	G513	28		18	10	**63**	**61**	**65**								
	L514	29		29	23	**60**	**63**	**56**								
	G515	29		40	33	50	53	**52**								
	L516	**58**		**65**	23	**81**	**75**								18	
	P517	**50**		50	21	**68**	**55**								9	
	L518	33		35	41	**66**	**62**								5	
	S519	**77**		**54**	34	**76**	**55**								**32**	
	P520	**41**		33	21	**64**	**58**								8	
	R521	11		26		**45**	**51**								5	
	L522					25	**48**									
	L523					35	**41**									
	P524					33	**45**									
	T525					44	31									
	A526					30										
	T527					27										
	T528															
	N529															
	G530															
	H531															
	P532															
	Q533															
	L534															
	P535															
	G536															
	H537															
	A538															
	K539															
	P540															
	G541															
	T542															
	P543															
	A544															
	L545															
	E546															
	T547															
	L548															
	E549															
	T550															
	T551															
	P552															
	P553															

Supplementary Fig. 7: Chemical crosslinking matrix. Average crosslinking yield in percent of total expressed arr2 derived from densitometric analysis of at least three independent experiments is shown (see Supplementary Data 2). Squares are colored according to crosslinking yield (color code in Fig. 2). Combinations with significant signal over control background noise (n≥3) are in bold. Combinations that did not yield crosslinking signal are shown in light blue. Other numbers indicate crosslinking signals not significantly different from control background noise (see Supplementary Fig. 8). Experiments without crosslinking signal were not quantified and are shown in light blue. Cysteine mutants of PTH1R positions in (a) the ICL1, ICL2, ICL3 and helix VIII, (b) the proximal, and (c) the distal C-terminus were combined with indicated BrEtY-arr2.

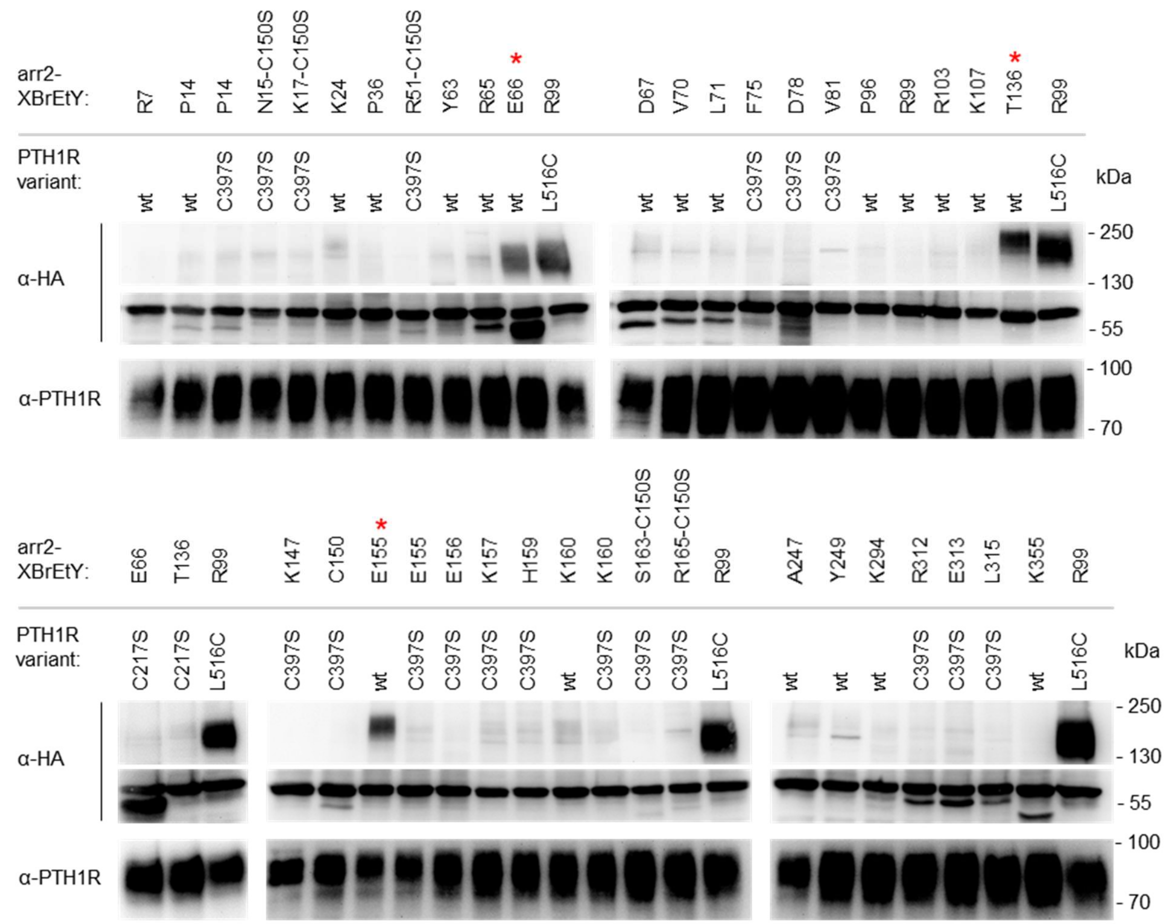

Supplementary Fig. 8: Chemical crosslinking background. Western blots of cell lysates stained with the antibodies indicated on the left of each panel are shown. Each of the 37 arrestin mutants giving crosslinking signals when combined with Cys-PTH1R mutants (screening results reported in Supplementary Fig. 6) were combined with either wt PTH1R or C397S-PTH1R, which gives the background for each combination. The background for BrEtY-arrestin that crosslinked wt-PTH1R was obtained by removing the involved Cys from the receptor (see Supplementary Figure 4), i.e. E66- and T136BrEtY-arr2 were combined with C217S-PTH1R; E155- and K160BrEtY-arr2 were combined with C397S-PTH1R. Arrestin mutants that crosslink with endogenous Cys in PTH1R are marked with a red asterisk. The combination of R99BrEtY-arr2 with L516C-PTH1R was included in each experiment as a positive control. When BrEtY was incorporated in the N-domain of arr2, the Cys150 of arr2 was mutated to Ser to avoid intramolecular quenching of BrEtY. Experiments were performed three times, signals were quantified by densitometry and used for the statistical analysis. Uncropped blots are provided as a Source Data file.

Supplementary Fig. 9. Crosslinking Experiments with arr2 variants lacking the finger loop.
Bpa was incorporated at the indicated positions in an arr2 variant where residues Y63 to K77 (finger loop) were deleted (ΔFLR-arr2). Bpa was incorporated at positions that gave chemical crosslinking with the C-tail of the PTH1R (Supplementary Fig. 6 and 7). These positions are located in the loop between β-strands I and II, and in the 160-loop of arr2. Cells co-expressing wt PTH1R and either ΔFLR-arr2 or full-length arr2 were treated with PTH(1-34) and irradiated with UV light (365 nm). We show the western blots analysis of the cell lysates (n=2) stained with an α-HA antibody. The numbering of arrestin positions is identical to wt.

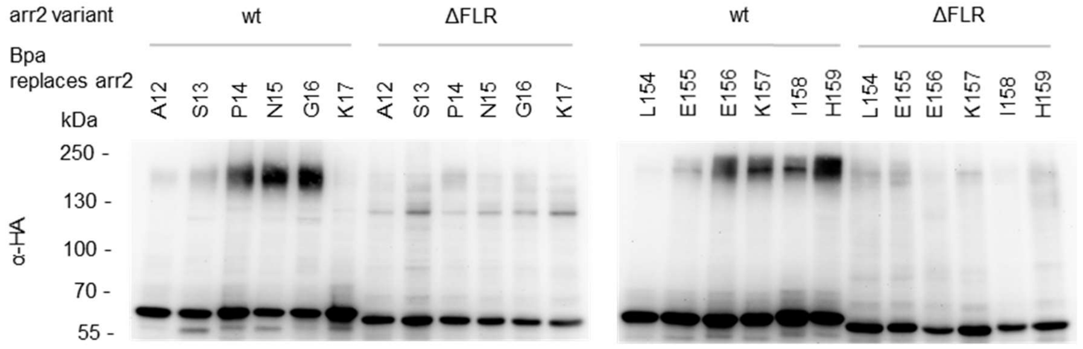

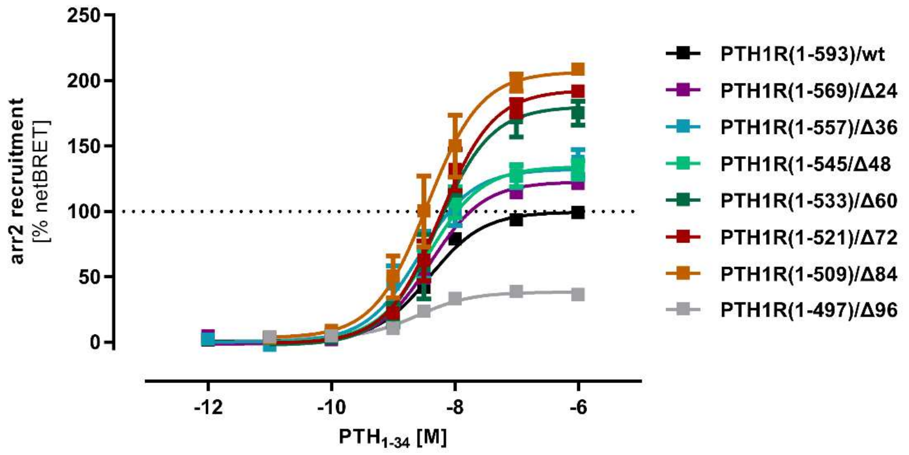

	1-593/wt	1-569/Δ24	1-557/Δ36	1-545/Δ48	1-533/Δ60	1-521/Δ72	1-509/Δ84	1-497/Δ96
$BRET_{max}$ ± s.e.m. [%]	100	123 ± 4	132 ± 7	134 ± 6	180 ± 10	193 ± 7	207 ± 11	38 ± 2
$BRET_{50}$ [nM]	4	4	3	4	5	6	4	1
95 % CI	3 – 5	3 – 5	1 – 5	2 – 6	3 – 9	4 – 8	2 – 6	0.1 – 4

Supplementary Fig. 10: Recruitment of arr2 by truncated variants of PTH1R. Dose-response curves. PTH1R was successively truncated in 12-residue steps and the C-terminus was directly fused to Nluc without a spacer. HEK293T cells expressing PTH1R-Nluc variants and VE-arr2 were stimulated with indicated concentrations of PTH(1-34). Luminescence and fluorescence were measured after 11 min. NetBRET values (fluorescence/luminescence) were normalized to the maximal netBRET of wt PTH1R. Plots show means ± SEM of at least four independent experiments (PTH1R(1-497) n=4, PTH1R(1-569) and PTH1R(1-545) n=5, PTH1R(1-557) and PTH1R(1-533) n=6, PTH1R(1-521) and PTH1R(1-509) n=7, and PTH1R(1-593) n=12), each performed in quadruplicates, the table shows the geometric mean of the $BRET_{50}$ values with 95% confidence intervals. Source data are provided as a Source Data file.

The intensity of the netBRET signal is inversely proportional to the distance between donor and acceptor. The gradual increase of netBRET with shortening of the receptor tail indicates a decreased distance between the luciferase and Venus. Note that when the phosphorylation site at S519 is removed as in PTH1R(1-509), arrestin recruitment is not affected. In contrast, when the distal phosphorylation cluster is deleted, as in PTH1R(1-497)/del96), arrestin recruitment is impaired, as reflected in the drastic drop of the $BRET_{max}$ from 207% to 38% of wild type signal. These results confirm the key role of the distal phosphorylation cluster and do not exclude an accessory role of the single phosphorylation site at S519.

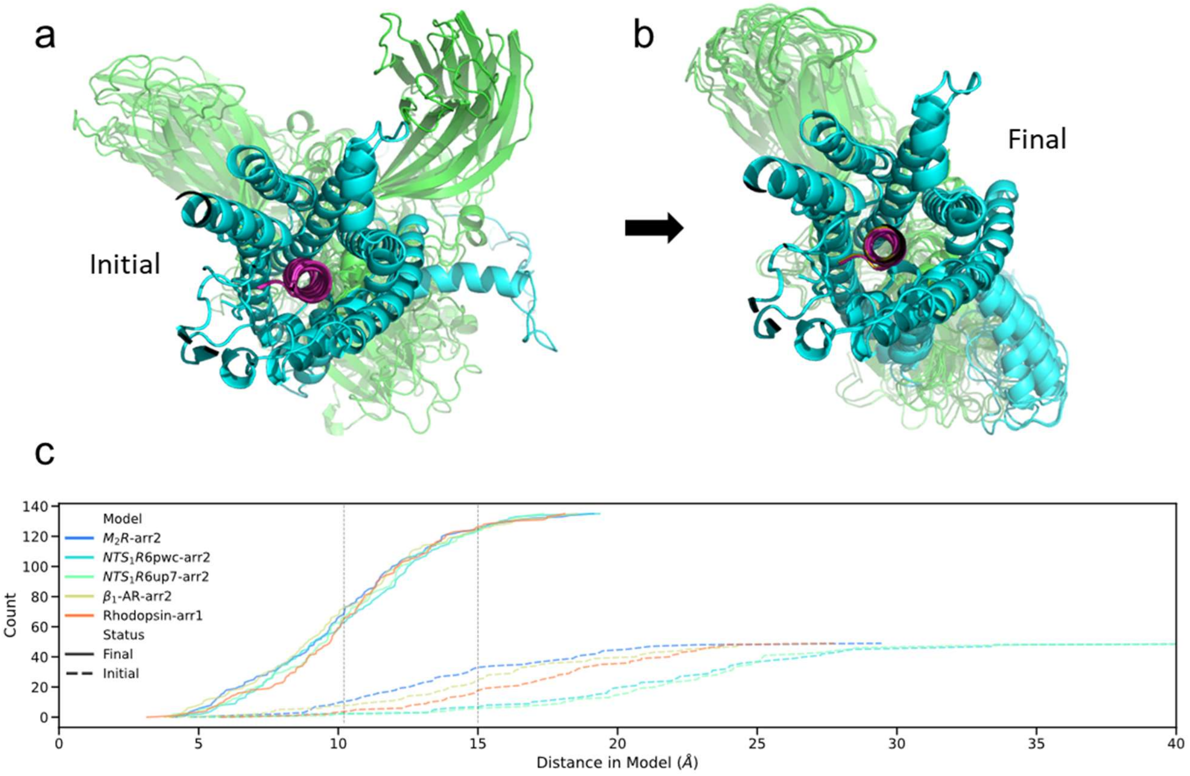

Supplementary Fig. 11: Performance of optimized models from each template. (a) Initial models superimposed at the receptor (b) Final models superimposed at the receptor. The conformational sampling converged to M_2R-arr2-like orientations of arrestin for all five templates (panel b). This holds true also for the two NTS_1R-arr2 templates, although in those initial structures the arrestin engages NTS_1R in an almost perpendicular orientation compared to its pose in the other three complexes as shown in panel (a).

(c) The cumulative distribution of intermolecular Cβ-Cβ distances in amino acids pairs that yielded pairwise crosslinks measured in different models. Dashed lines show the distribution of distances in initial models, solid lines show the distribution of distances after optimization. In the initial models, crosslinks involving C-termini (res. 483-525) in PTH1R are excluded as they are not covered by structural templates. These results show that, while the initial models vary in their quality, the five final models showed very similar improved distributions with the M_2R-arr2–based model showing the best fit to the experimental data.

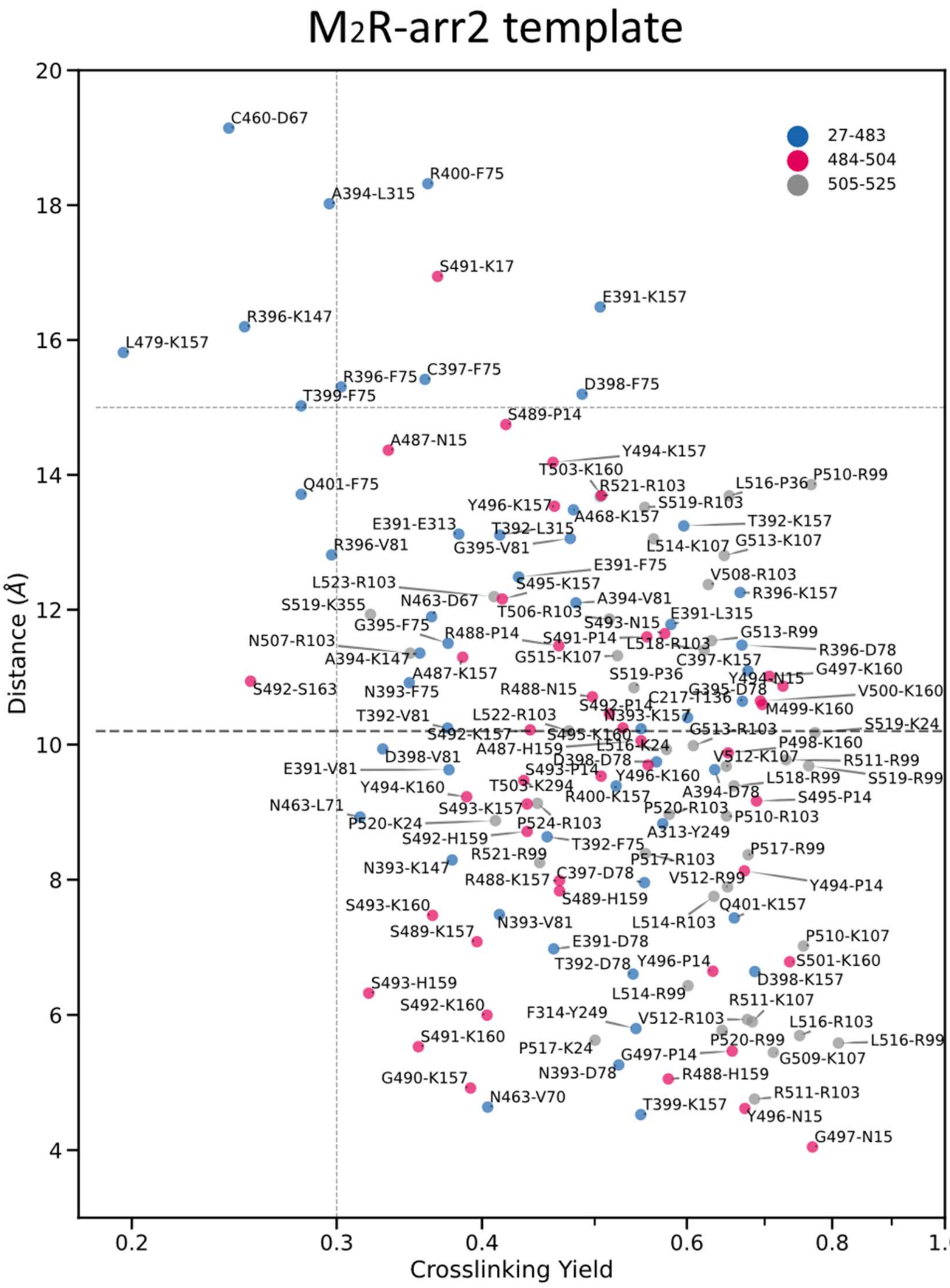

Supplementary Fig. 12: Plot of pairwise Cβ-Cβ distances against chemical crosslinking yield in an optimized PTH1R-arr2 model after flexible optimization starting from a M_2R-arr2 (PDBID: 6u1n) template; when glycine is present, Cα was used instead. Color code corresponds with PTH1R residue index. The horizontal dashed line at 10.2 Å marks the estimated (Cβ-Cβ) distance for BrEtY-Cys crosslinking, whereas the dotted line at 15.0 Å represents the maximal crosslinking distance when considering the flexibility of the complex. The vertical line marks 30% crosslinking yield. The data presented here are the same as Figure 3a in the main text; we include this for completeness.

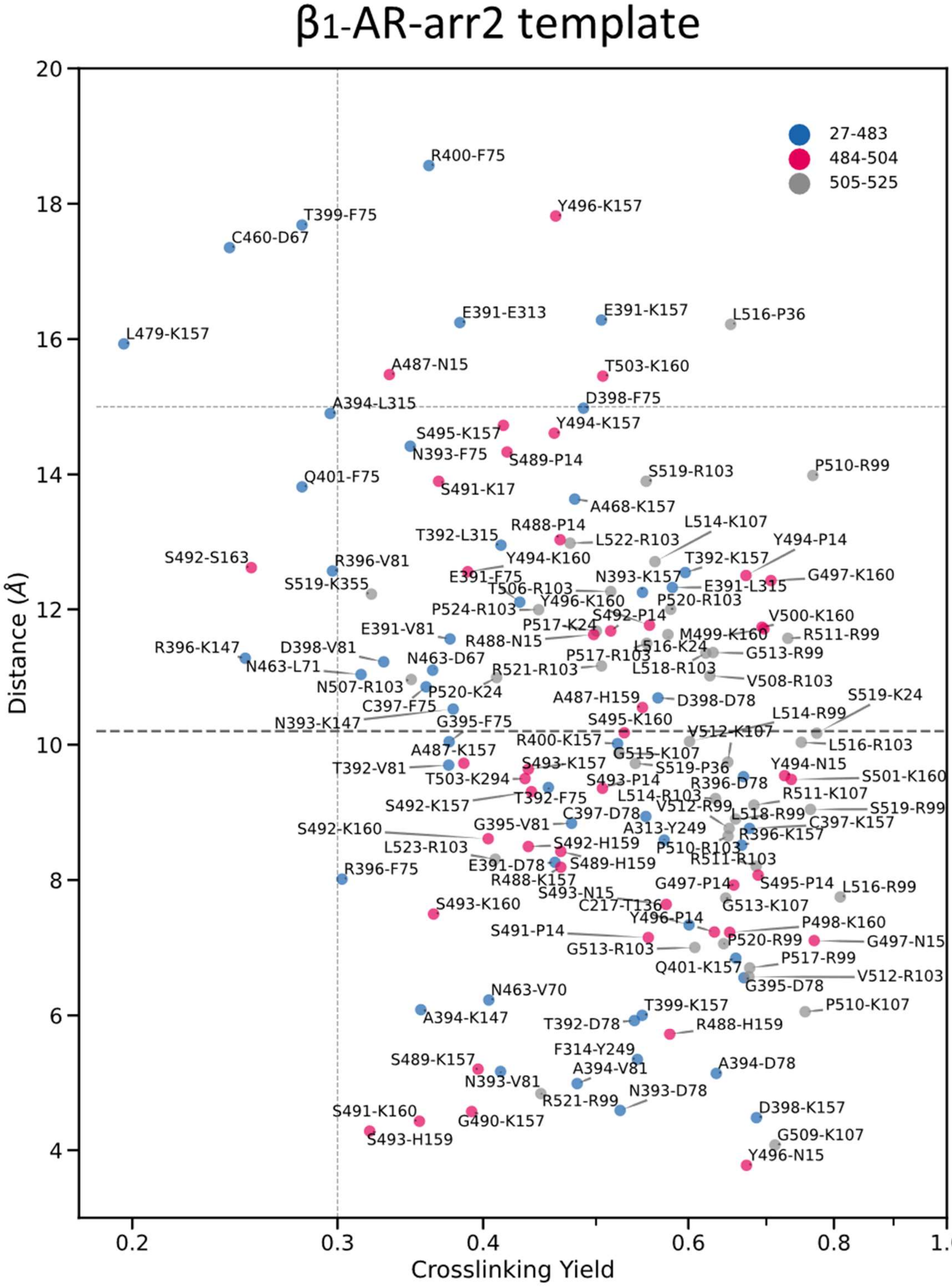

Supplementary Fig. 13: Plot of pairwise Cβ-Cβ distances against chemical crosslinking yield in an optimized PTH1R-arr2 model after flexible optimization starting from a β_1-AR-arr2 (PDBID: 6tko) template; in case when glycine is present, Cα is used instead. Color code corresponds with PTH1R residue index. The horizontal dashed line at 10.2 Å marks the estimated (Cβ-Cβ) distance for BrEtY-Cys crosslinking, whereas the dotted line at 15.0 Å represents the maximal crosslinking distance when considering the flexibility of the complex. The vertical line marks 30% crosslinking yield.

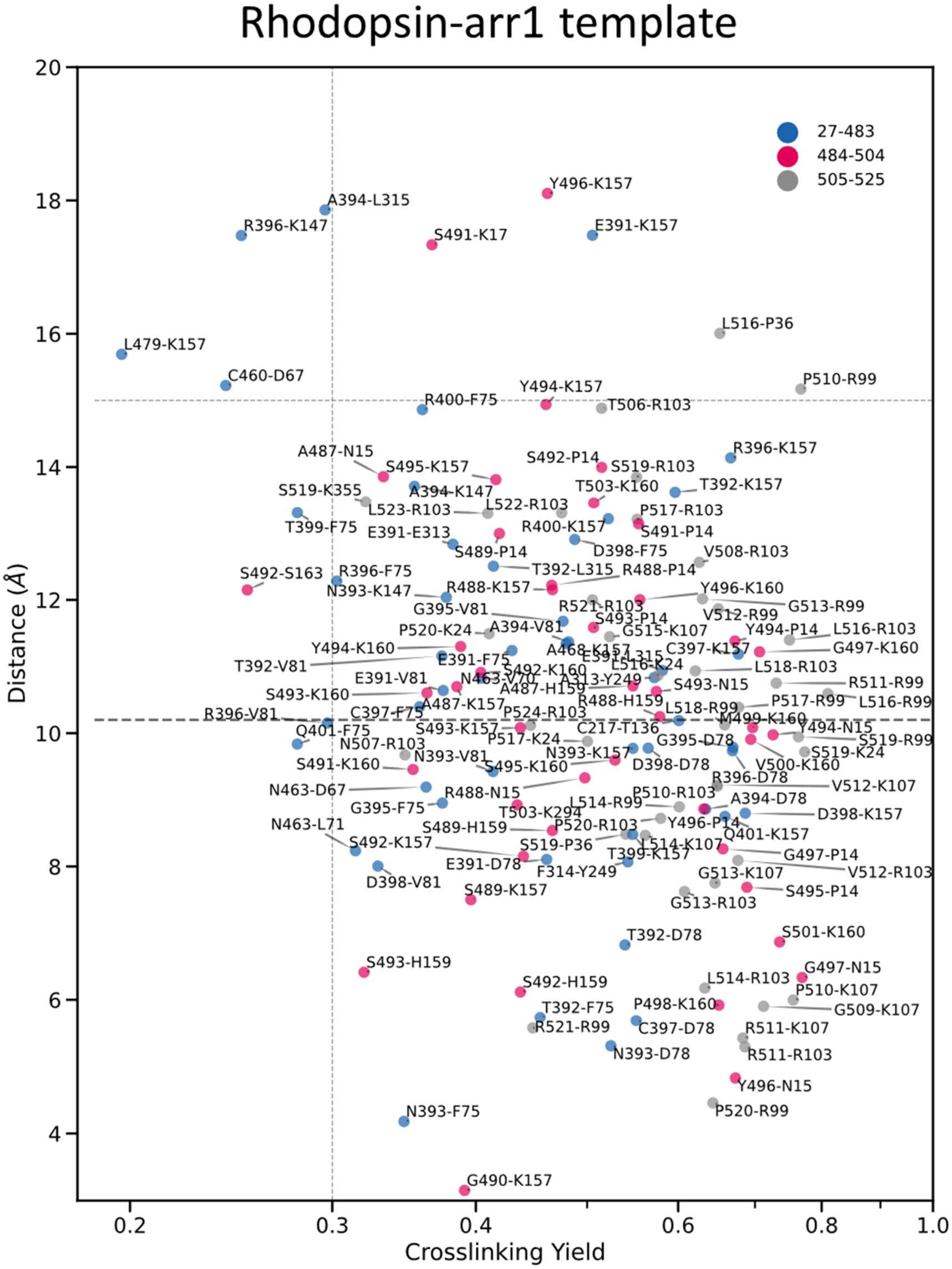

Supplementary Fig. 14: Plot of pairwise Cβ-Cβ distances against chemical crosslinking yield in an optimized PTH1R-arr2 model after flexible optimization starting from a Rhodopsin-arr1 (PDBID: 5w0p) template; when glycine was present, Cα was used instead. Color code corresponds to PTH1R residue index. The horizontal dashed line at 10.2 Å marks the estimated (Cβ-Cβ) distance for BrEtY-Cys crosslinking, whereas the dotted line at 15.0 Å represents the maximal crosslinking distance when considering the flexibility of the complex. The vertical line marks 30% crosslinking yield.

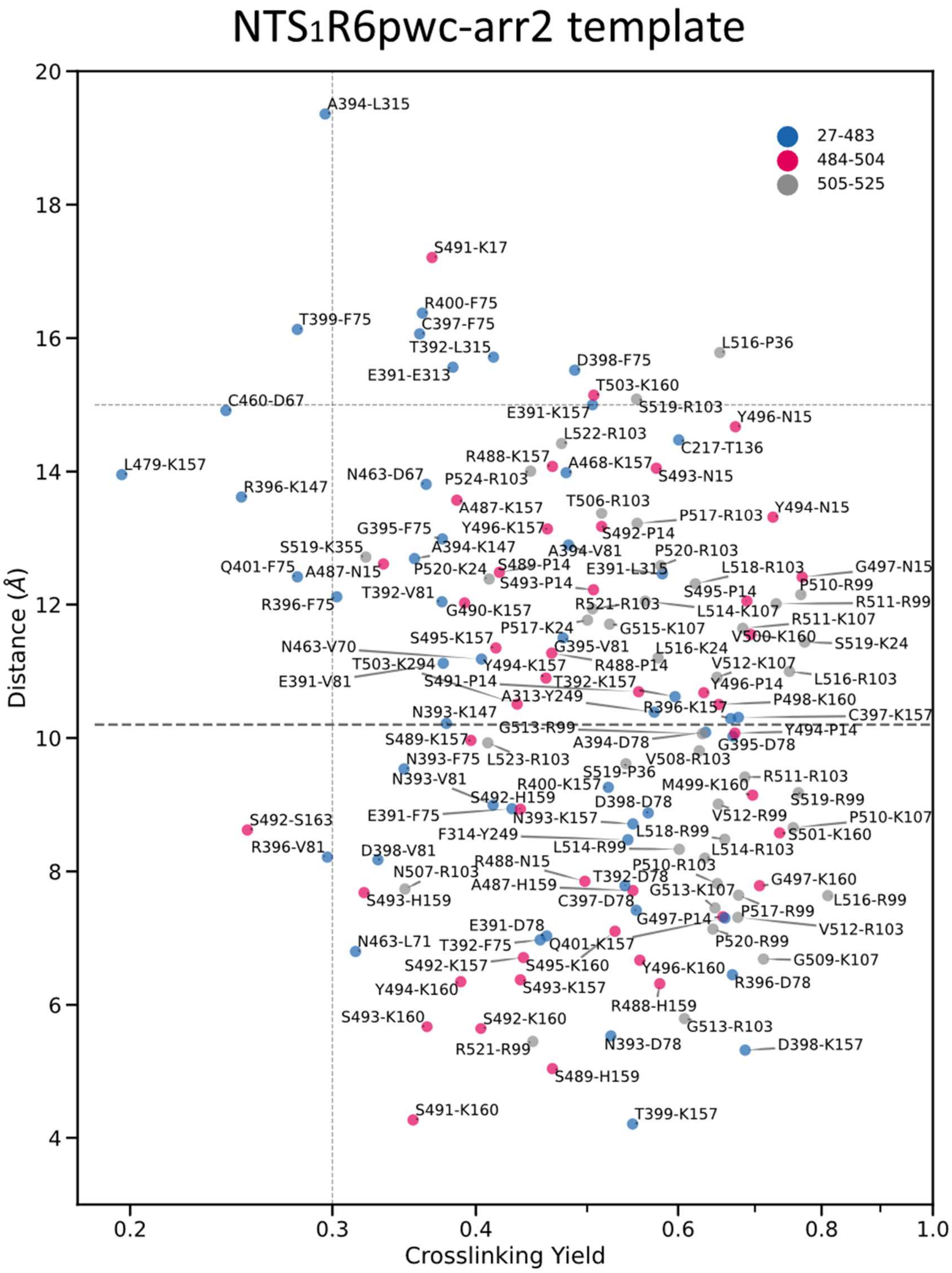

Supplementary Fig. 15: Plot of pairwise Cβ-Cβ distances against chemical crosslinking yield in an optimized PTH1R-arr2 model after flexible optimization starting from a NTS_1R-arr2 (PDBID: 6pwc) template; when glycine was present, Cα was used instead. Color code corresponds to PTH1R residue index. The horizontal dashed line at 10.2 Å marks the estimated (Cβ-Cβ) distance for BrEtY-Cys crosslinking, whereas the dotted line at 15.0 Å represents the maximal crosslinking distance when considering the flexibility of the complex. The vertical line marks 30% crosslinking yield.

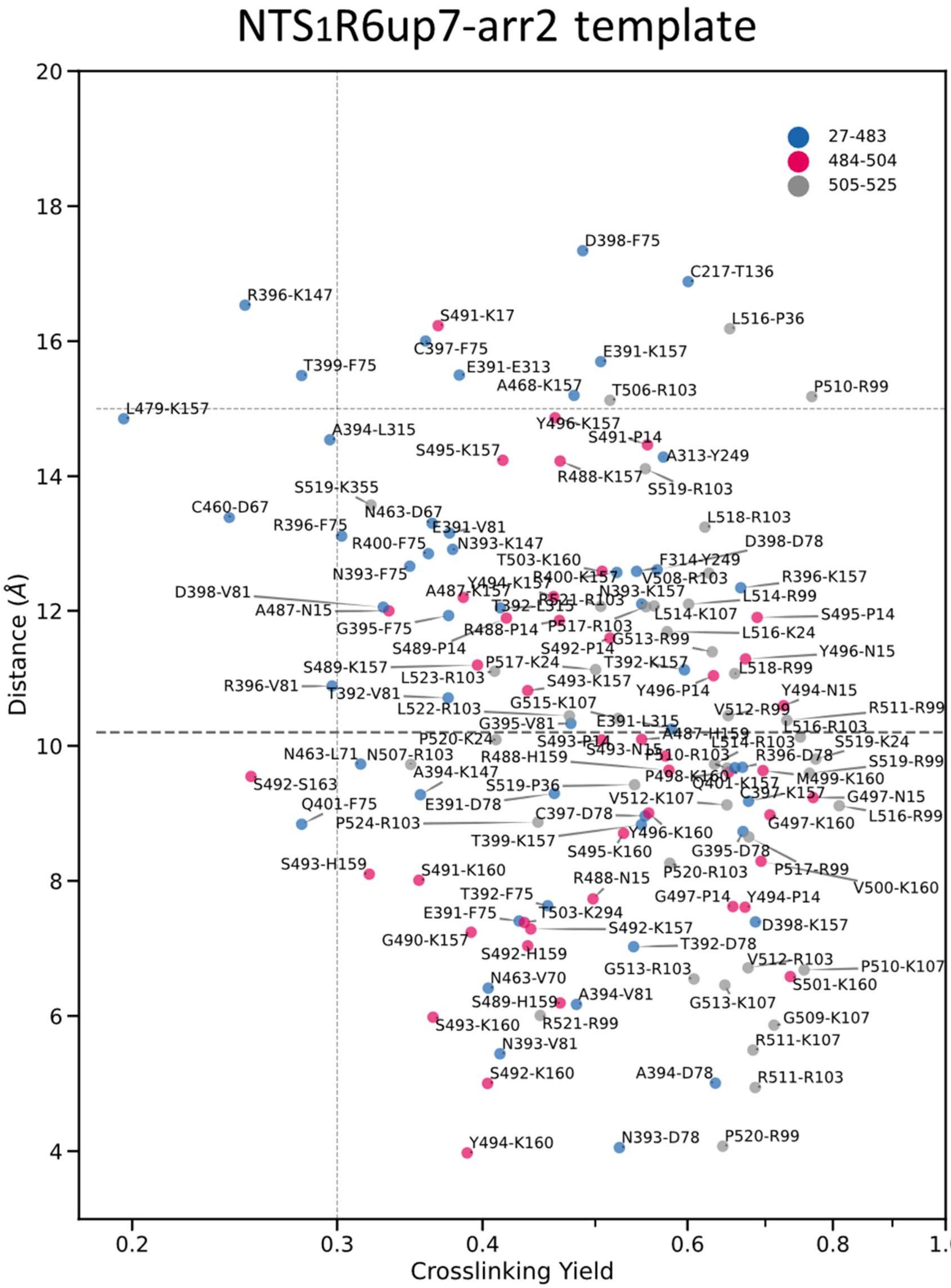

Supplementary Fig. 16: Plot of pairwise Cβ-Cβ distances against chemical crosslinking yield in an optimized PTH1R-arr2 model after flexible optimization starting from a NTS_1R-arr2 (PDBID: 6up7) template; when glycine was present, Cα was used instead. Color code corresponds to PTH1R residue index. The horizontal dashed line at 10.2 Å marks the estimated (Cβ-Cβ) distance for BrEtY-Cys crosslinking, whereas the dotted line at 15.0 Å represents the maximal crosslinking distance when considering the flexibility of the complex. The vertical line marks 30% crosslinking yield.

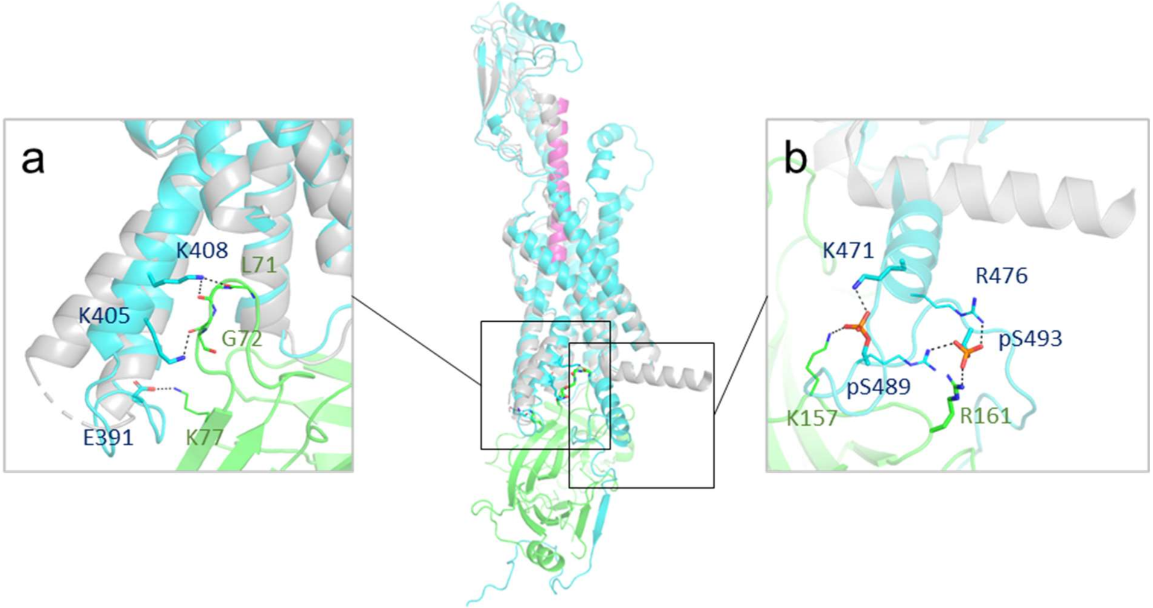

Supplementary Fig. 17: Comparison of the PTH1R conformation in a G protein-bound complex with our optimized model of the PTH1R-arr2 complex. The cryo-EM structure of G_s-bound PTH1R (PDBID: 6nbf) in light grey cartoon is superposed to our model in cyan/green/magenta. (a) Inward adjustment of ~2Å at TM6/ICL3 of PTH1R in our model based on the M_2R-arr2 template after flexible refinement. The interaction between PTH1R and arr2 is supported by various polar/ionic contacts around the region, which also persisted in unbiased MD simulations (see SI fig. 22). The inward movement of TM5/6 in our model is in line with the observation made in other arrestin-bound experimental structures that the TM5/6 are slightly closer (drawn inward to the cleft) compared to the respective G-protein bound complexes. (b) Movement of helix VIII in a network of interaction with the proximal cluster.

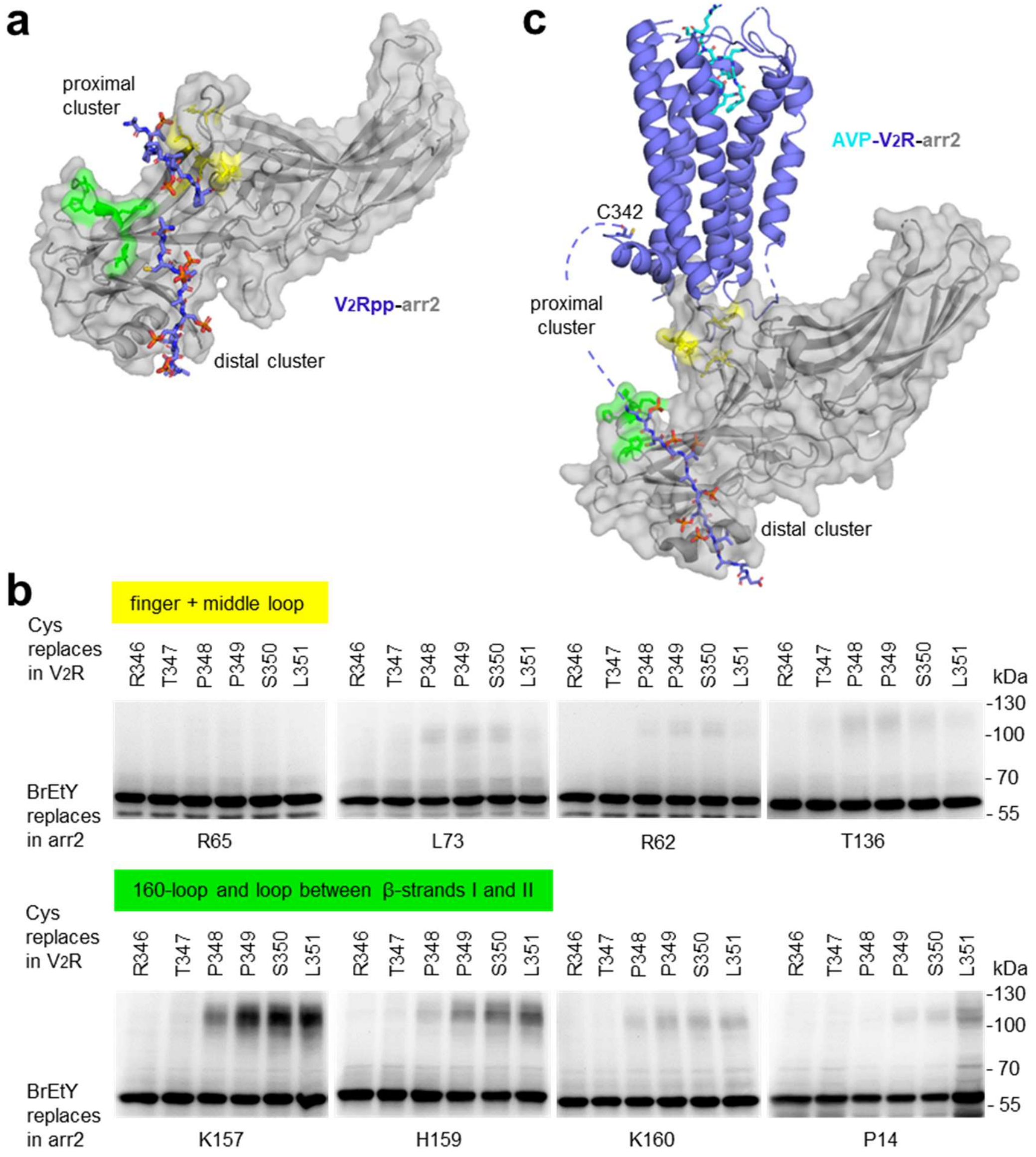

Supplementary Fig. 18: Position of proximal cluster in the V_2R on arr2.

a) Crystal structure of arr2 bound to the V_2Rpp (PDBID: 4jqi). Arrestin is shown in grey as cartoon and surface, V_2Rpp as purple sticks with phosphates in orange. The positions of the proximal (^{347}pTPPpS^{350}) and the distal phosphorylation cluster (^{357}pSCpTpTApSpSpS^{364}) of V_2Rpp are indicated. b) Western blots of cell lysates (n=1) from pairwise crosslinking at full-length V_2R, stained with an α-HA antibody. Cys mutations in the proximal cluster of V_2R are indicated on top of each lane. To avoid interference by C142, C358 of V_2R, these were mutated to Ala. Residues of arr2 substituted by BrEtY are indicated below the blot and highlighted in the V_2Rpp (panel a) and in full-length V_2R (panel c) in yellow and green. c) Cryo-EM structure of arr2 bound to the full-length V_2R (PDBID: 7r0c). Arrestin is shown in grey as cartoon and surface, V_2R 7TM is shown as purple cartoon, and V_2R C-terminus is shown as sticks with phosphates in orange. The position of the distal phosphorylation cluster of V_2R is indicated, the proximal cluster is not resolved.

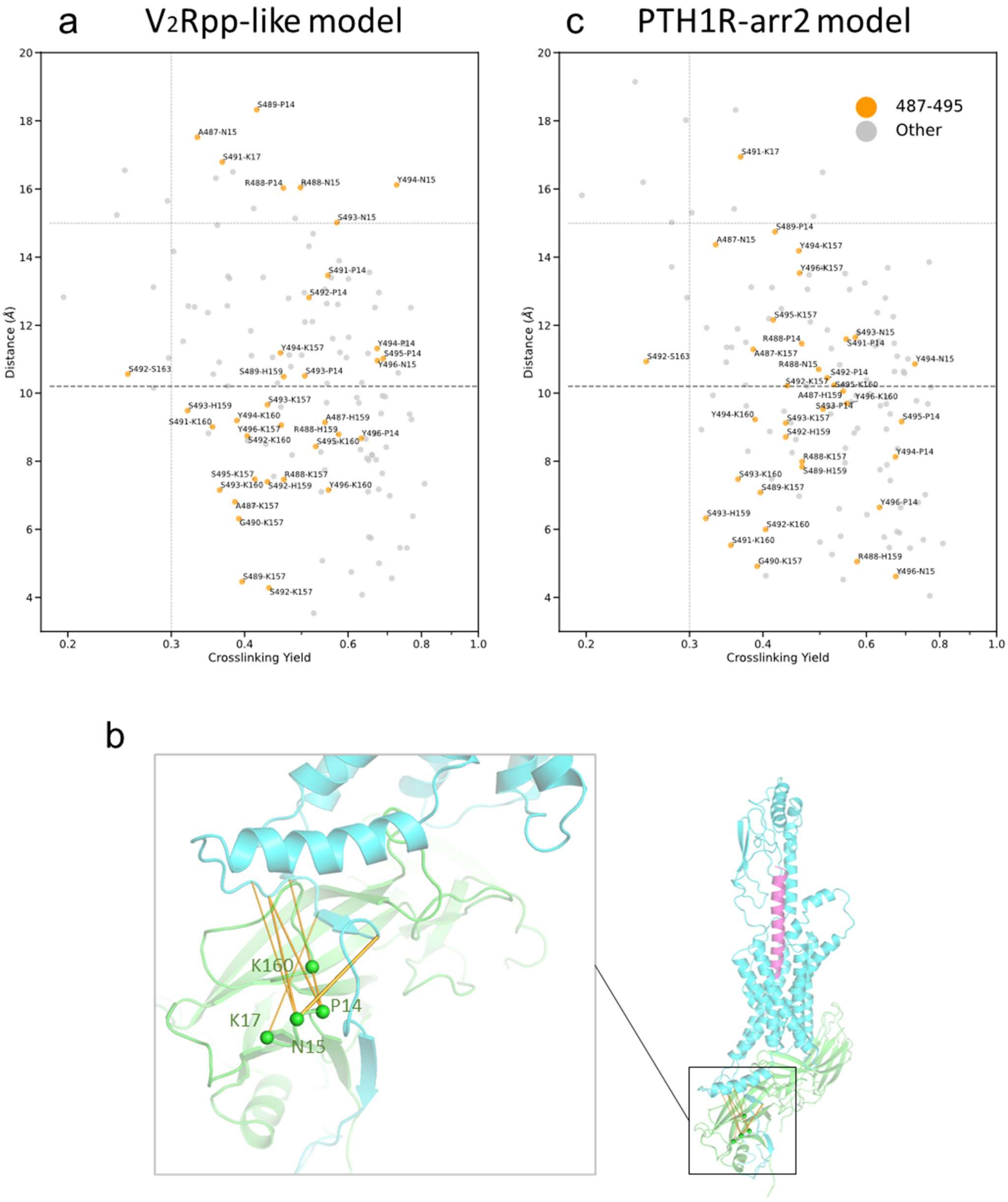

Supplementary Fig. 19. Validation of the N-edge path of the C-tail: analysis of a model forcing the interaction of the proximal phosphorylation cluster with the finger loop. To test whether the path of the proximal phosphorylation cluster observed for the V_2R phosphopeptide (V_2Rpp) bound to arr2 (PDBID: 4jqi) is reconcilable with our crosslinking data, we built a V_2Rpp-like PTH1R-arr2 model based on our crosslinking

dataset with 3 additional distance restraints between residues 489-493 of PTH1R (proximal cluster) and residues 74-76 of arr2 (β-strand VI, just downstream of the finger loop), which are meant to force β-strand formation between the proximal cluster and the β-strand VI as observed in PDBID: 4jqi. The restrains are flat-bottomed potentials with walls at 2.5 Å and 5.0 Å and 30 times stronger than a distance restraint at the maximal crosslinking yield $B_{ij} = 1$. This V_2Rpp-like PTH1R-arr2 model was built from the M_2R-arr2 template (PDBID: 6u1n) using the same flexible optimization protocol as all our other models.

Panels a and c are plots of Cβ-Cβ distances measured on the models against experimental yields of chemical crosslinking at the represented amino acid pairs. Note that panel c represents the same data as in Supplementary Figure 12, just with different colors. The horizontal dashed line at 10.2 Å marks the estimated (Cβ-Cβ) distance for BrEtY-Cys crosslinking, whereas the dotted line at 15.0 Å represents the maximal crosslinking distance when considering the flexibility of the complex. The vertical line marks 30% crosslinking yield. In both panel a and c, crosslinks involving the proximal cluster (res. 487-495) are highlighted in orange.

(a) The V_2Rpp-like model features overall 14 outliers out of the crosslinking range (grey and orange dots above the 15 Å line). Of these, seven involve the stretch 487-494 of PTH1R including the proximal phosphorylation cluster (orange dots). Among the 14 outliers, 11 featured crosslinking yields >0.3 (grey and orange dots right of the vertical line).

(b) A zoom-in on V_2Rpp-like model. The seven crosslinks with >15 Å Cβ-Cβ distance involving the proximal cluster are shown as orange lines.

(c) Our best PTH1R-arr2 model features overall 11 outliers out of the crosslinking range (grey and orange dots above the 15 Å line), 6 featuring crosslinking yields >0.3 (grey and orange dots right of the vertical line). Of these, only one involves the stretch 487-494 of PTH1R including the proximal phosphorylation cluster (orange dot).

Overall, these data show that a V_2Rpp-like conformation has dramatically increased the level of strain in the proximal cluster region (7 vs. 1 outlier) and worsened the quality of the overall model (14 vs. 11 outliers, 11 vs. 6 at high crosslinking yield).

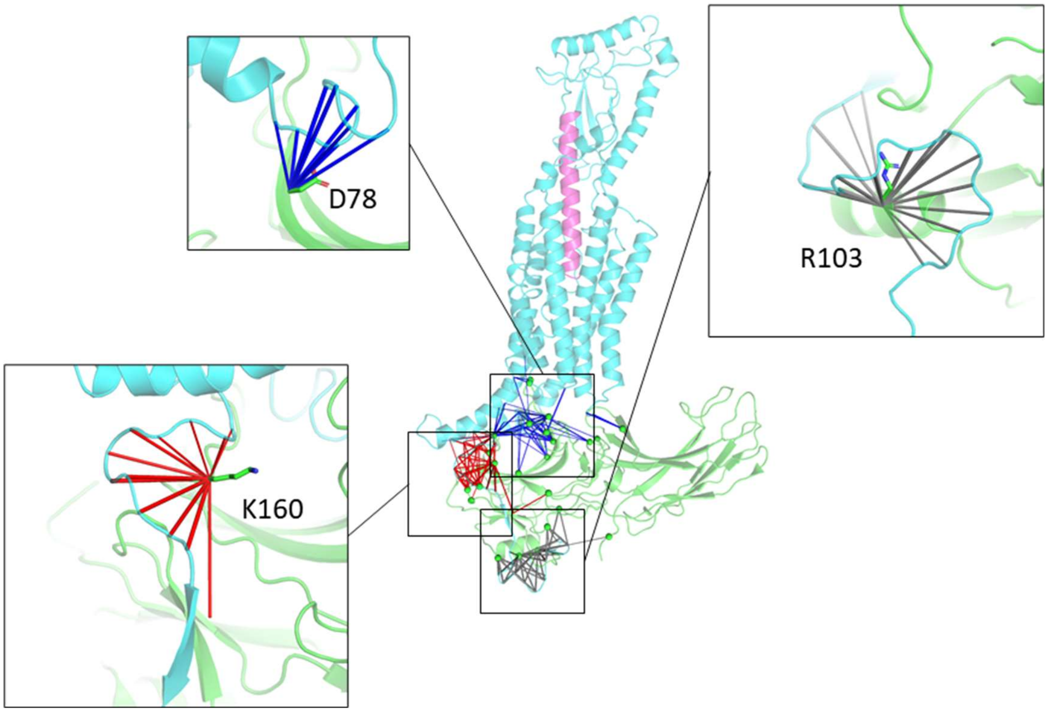

Supplementary Fig. 20 Examples of "crosslinking hubs" in different regions of arr2. BrEtY incorporated at position D78, R103 and K160 of arr2 (green stick representation) show significant pairwise crosslinking with 8, 17 and 12 different Cys-PTH1R mutants respectively. The pairings are indicated by red lines. Apparently not all these pairs can be accommodated concurrently due to steric "overcrowding".

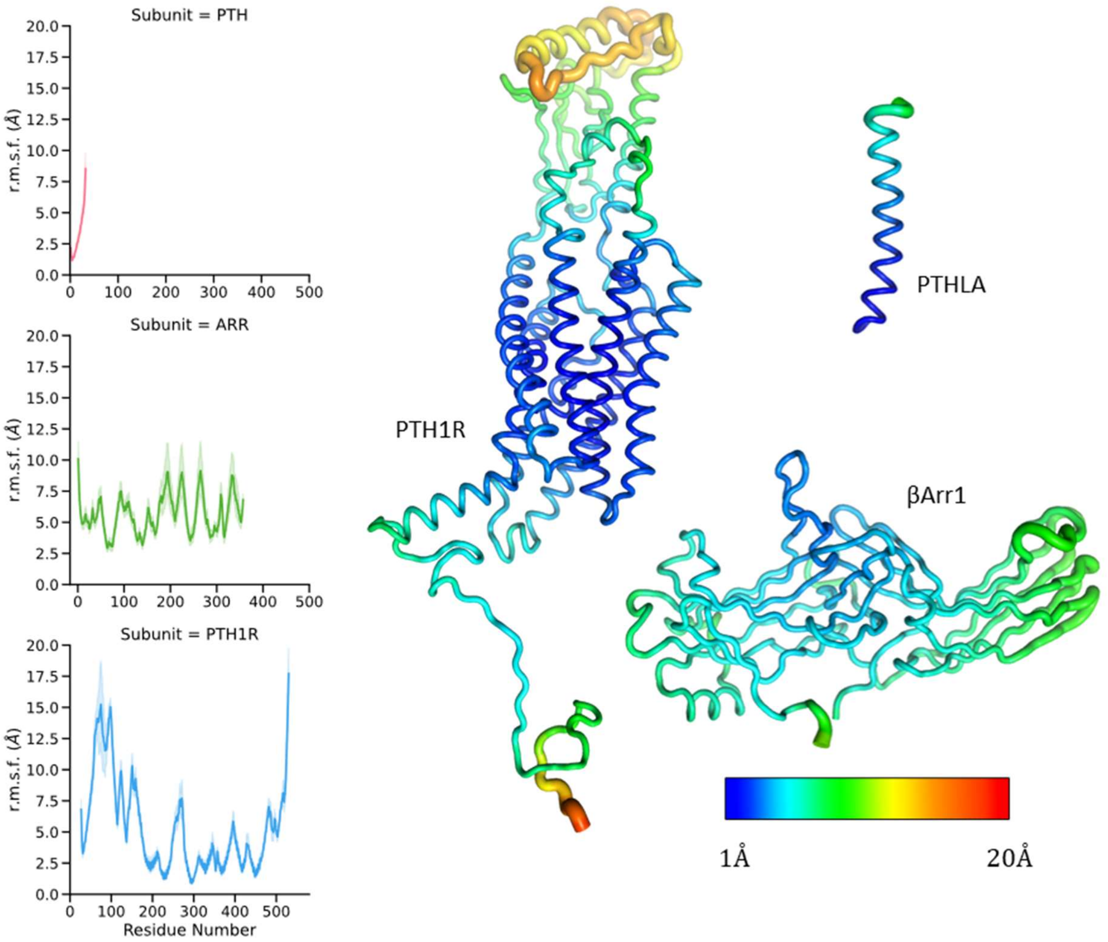

Supplementary Fig. 21: Root-mean-square fluctuation (r.m.s.f.) in MD simulations. Protein structures are color coded, as indicated.

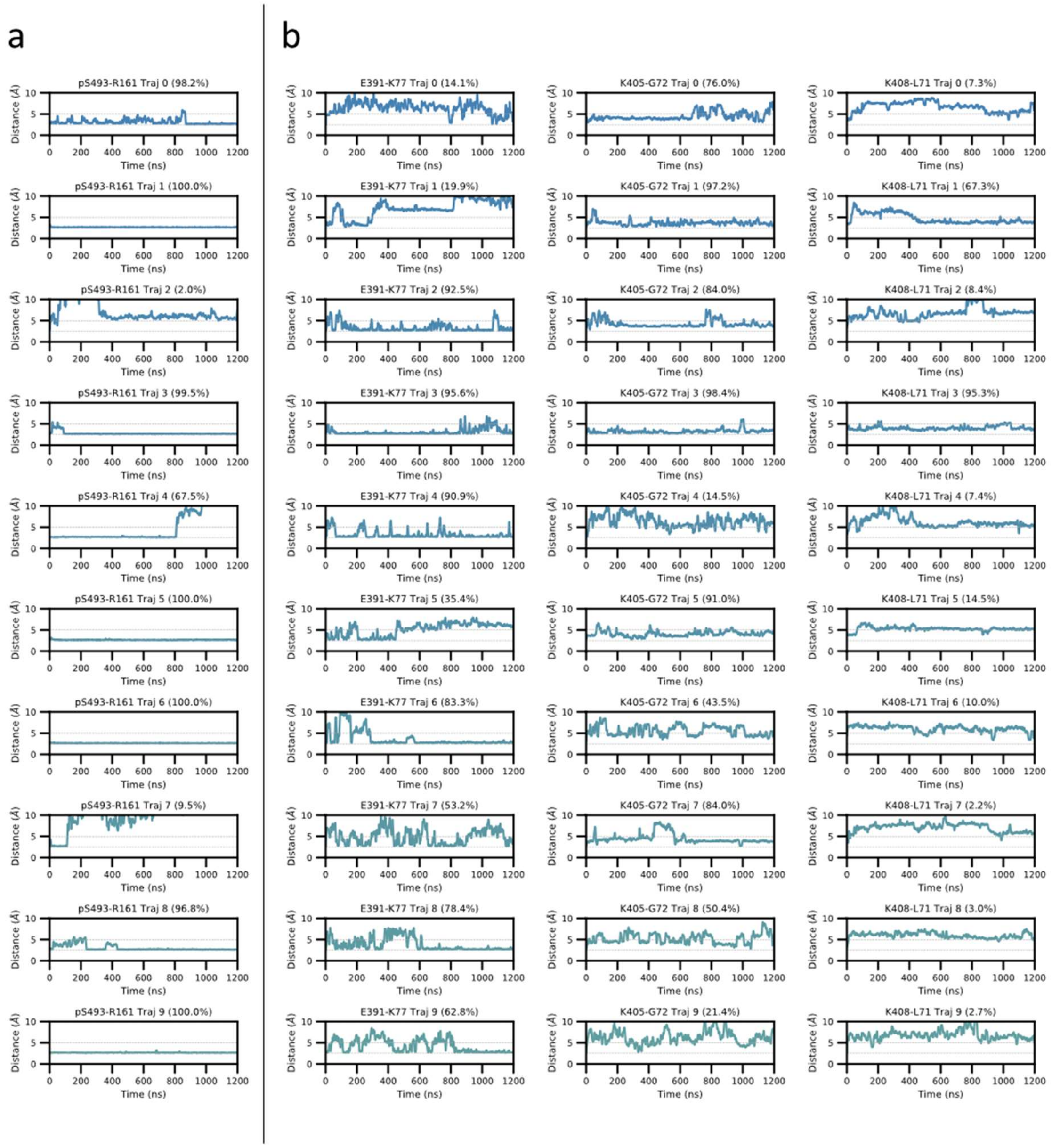

Supplementary Fig. 22: Interactions in MD Simulations. (a) between the proximal cluster of PTH1R and R161 on N-edge of arr2 (b) between finger loop of arr2 and TM6/ICL3 of PTH1R. The percentage of time where residues are within 5 Å is indicated in parenthesis for each trajectory.

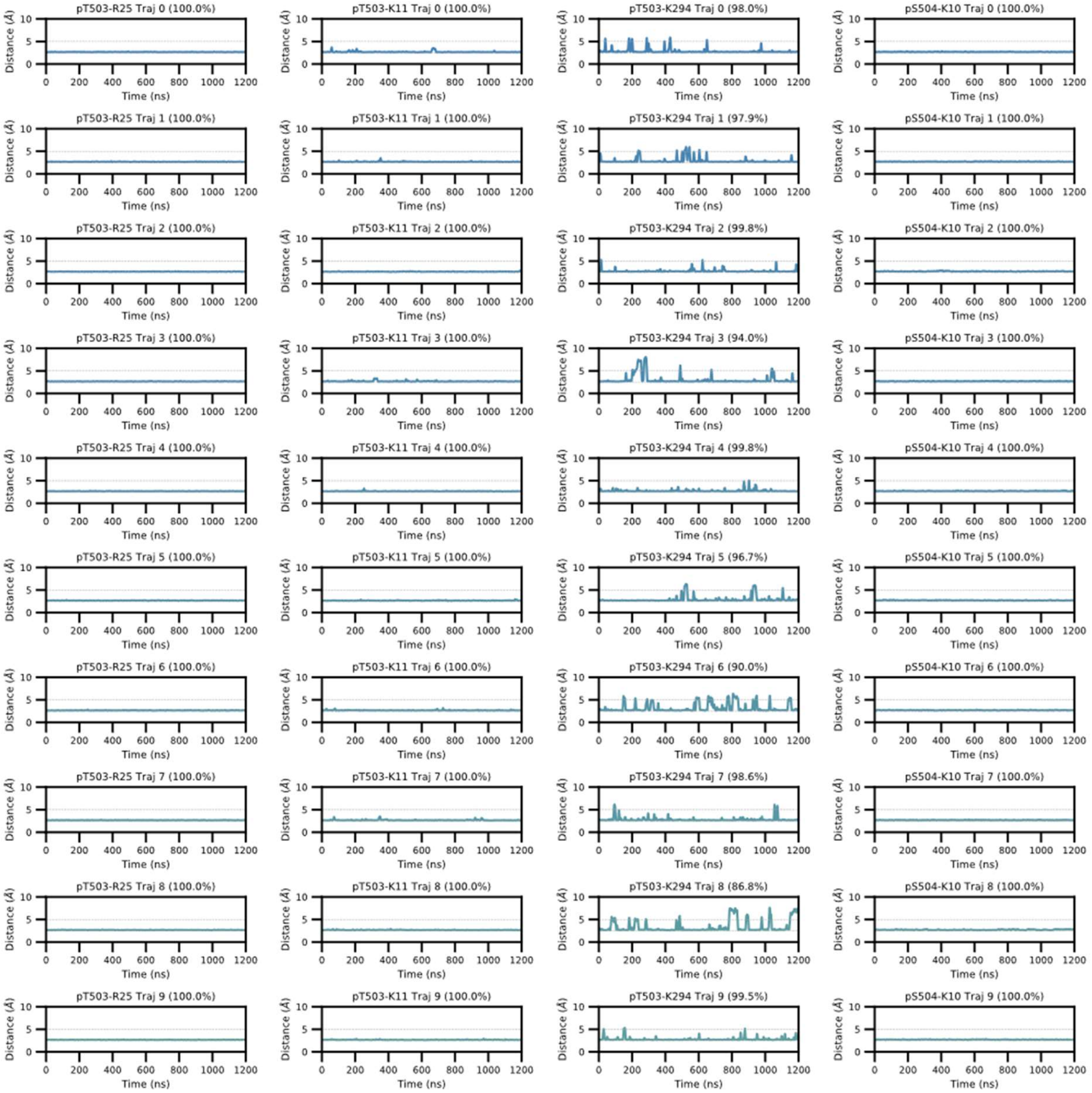

Supplementary Fig. 23: Interactions in MD Simulations. Between distal cluster of PTH1R and β-strand I of arr2. The percentage of time where residues are within 5 Å is indicated in parenthesis for each trajectory.

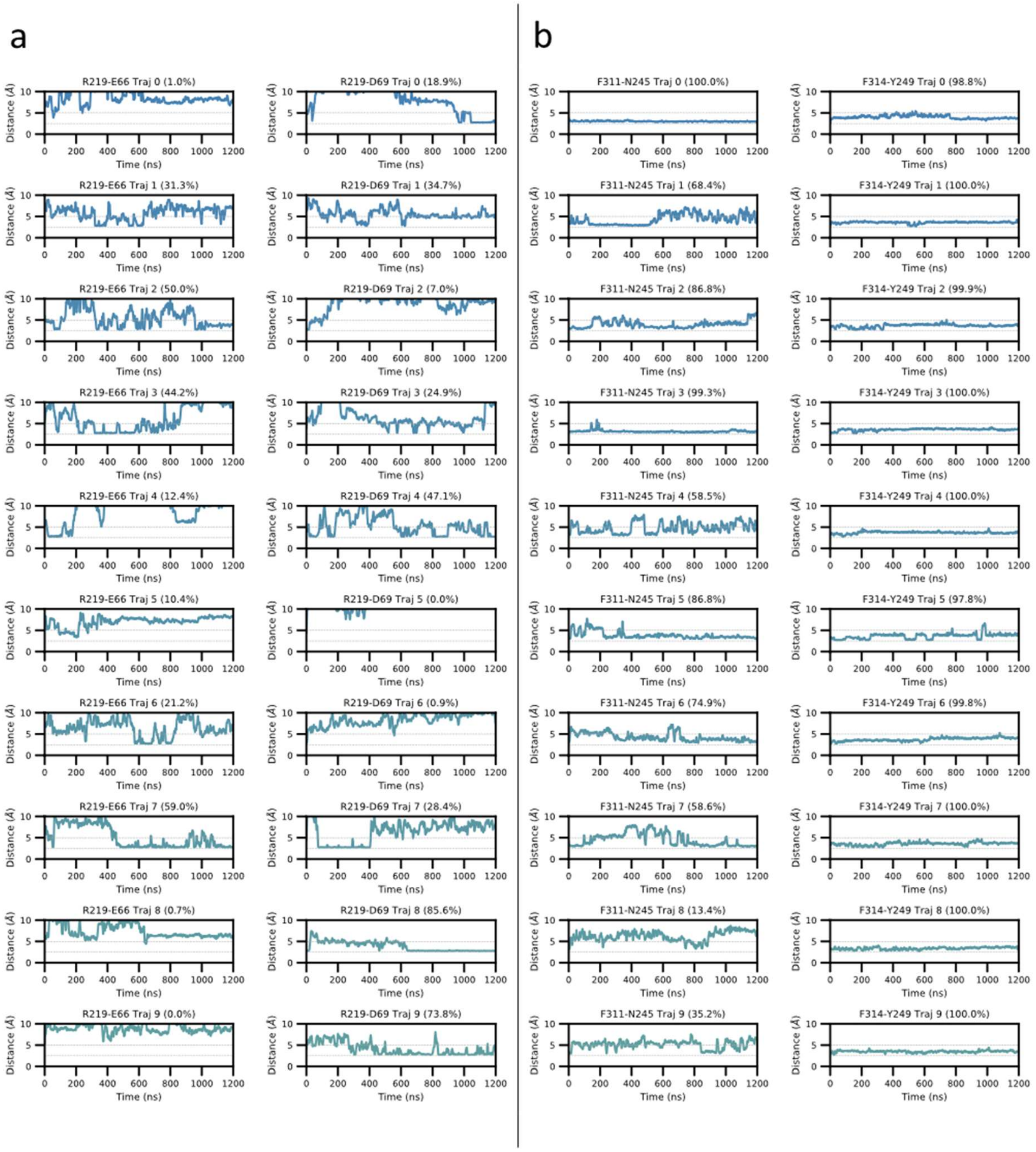

Supplementary Fig. 24: Interactions in MD Simulations. (a) between finger loop of arr2 and TM2 of PTH1R (b) between C-loop of arr2 and ICL2 of PTH1R. The percentage of time where residues are within 5 Å is indicated in parenthesis for each trajectory.

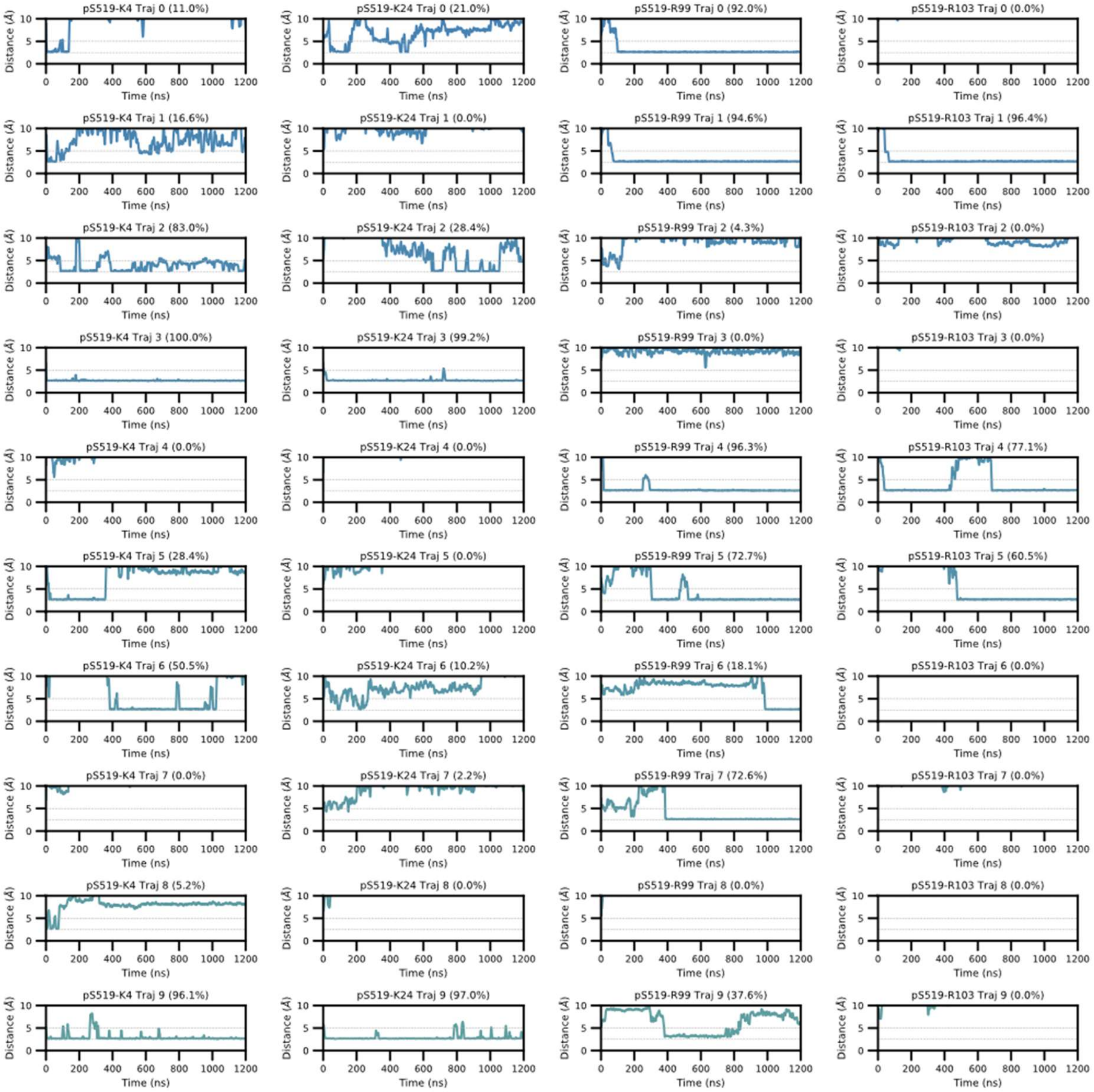

Supplementary Fig. 25: Interactions in MD Simulations. pS519 at the C-terminus of PTH1R and R99 of arr2. In some cases, the trajectory distance goes out of range of interest. The percentage of time where residues are within 5 Å is indicated in parenthesis for each trajectory.

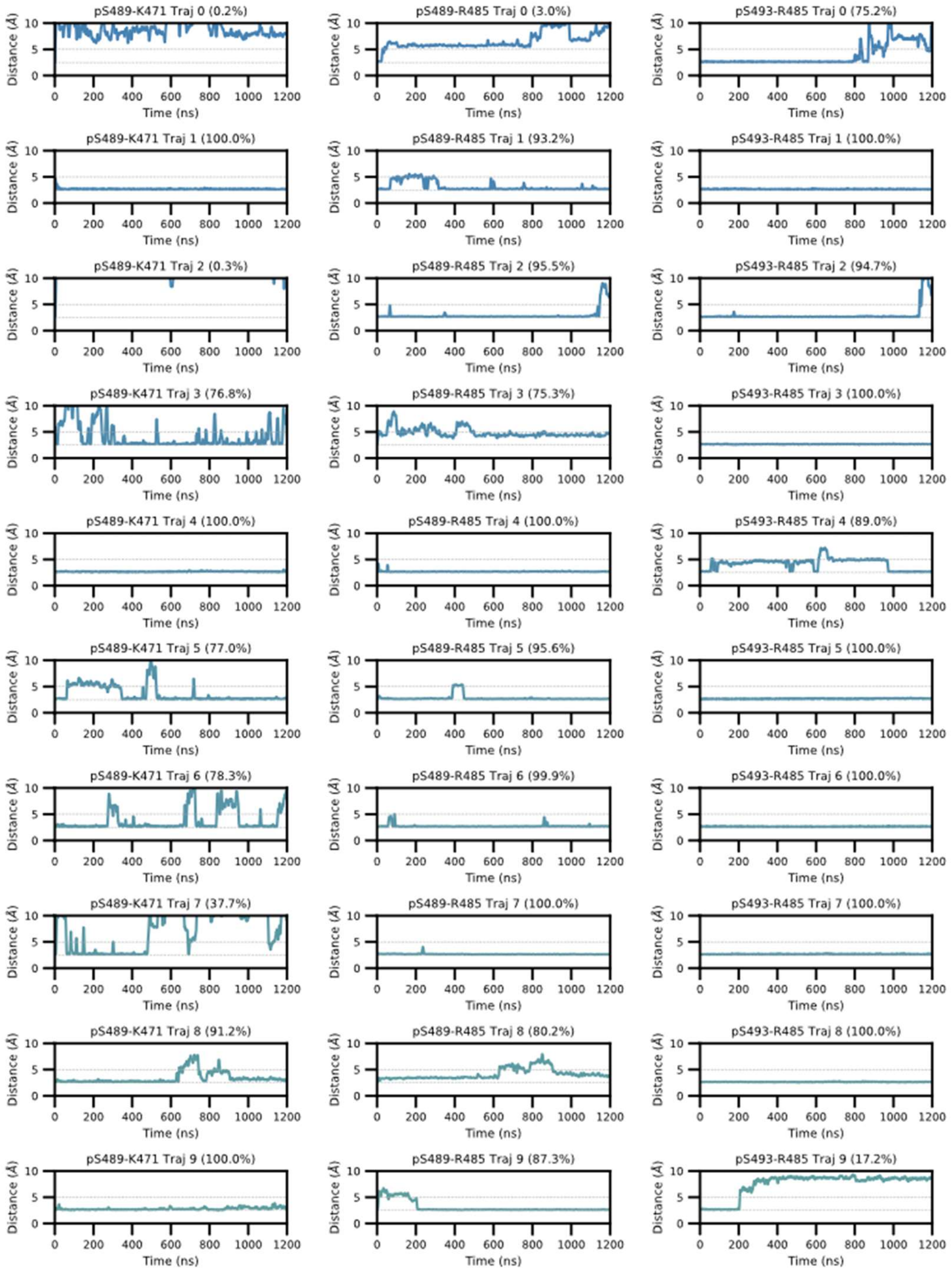

Supplementary Fig. 26: Interactions in MD Simulations. Interaction between proximal cluster and helix VIII. In some cases, the trajectory distance goes out of range of interest. The percentage of time where residues are within 5 Å is indicated in parenthesis for each trajectory.

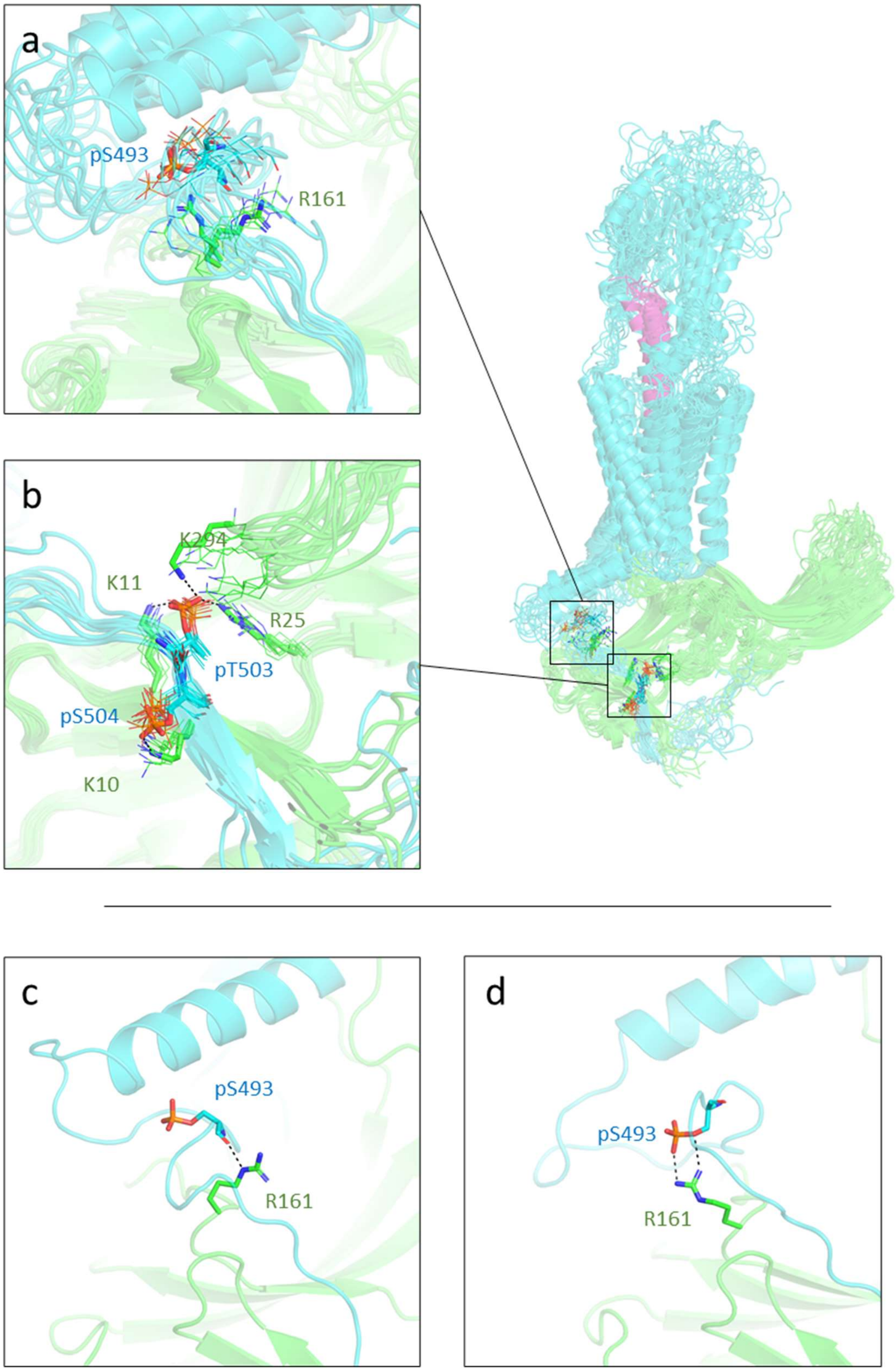

Supplementary Fig. 27: Proximal and distal phosphorylation clusters in MD simulation of the model refined from M_2R-arr2. Stacked snapshots of PTH1R proximal (a) and distal (b) phosphorylation cluster in one of the trajectories. Snapshots were taken every 100 ns spanning 1200 ns. (c,d) Two modes of interactions between pS493 of proximal cluster and R161 on the N-edge.

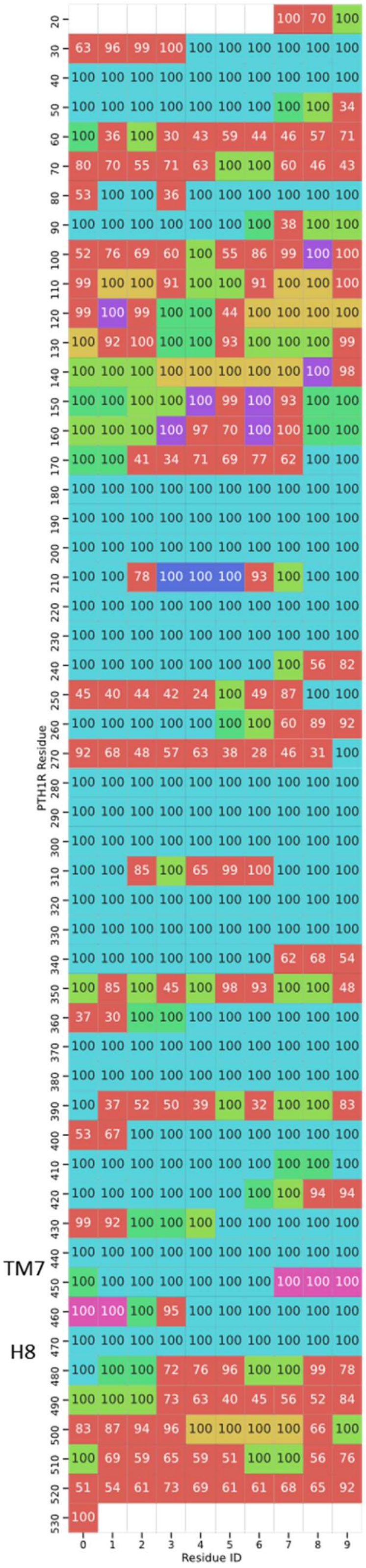

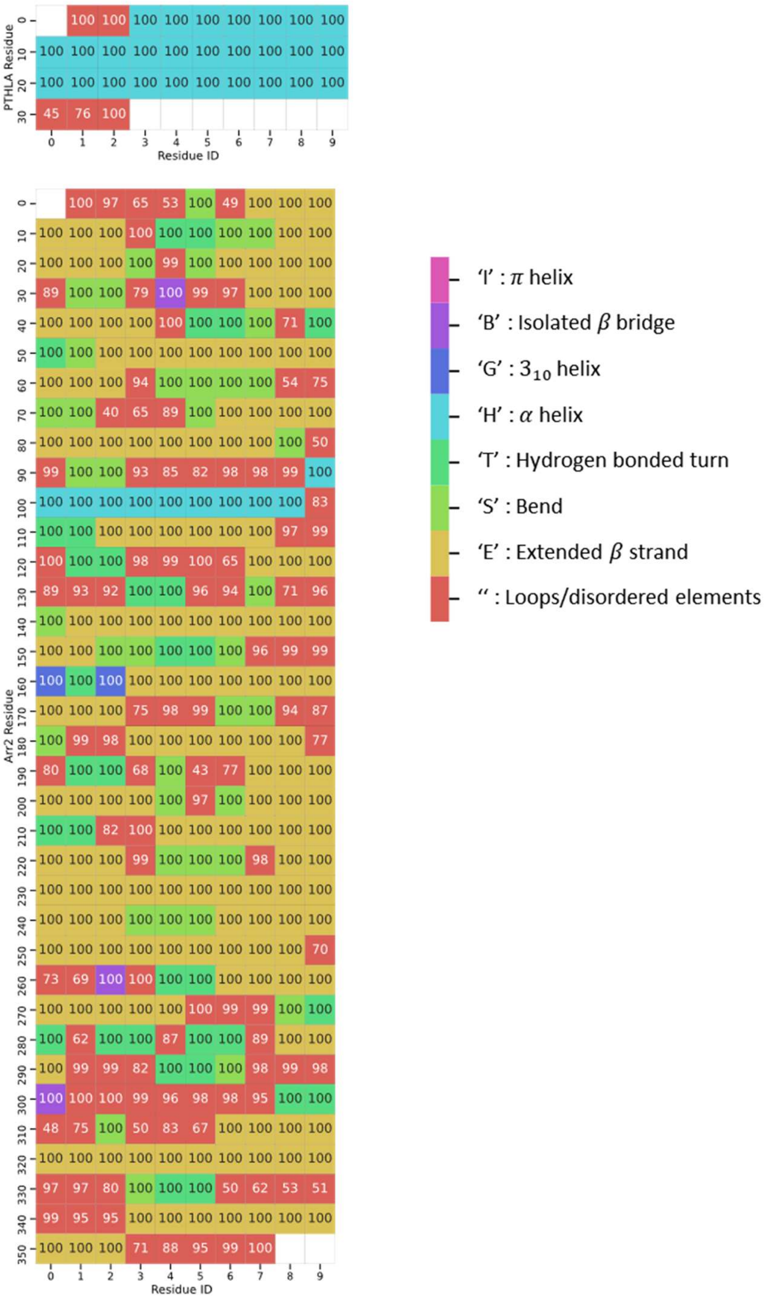

Supplementary Fig. 28: Secondary Structure Classification in MD simulations. We labeled each protein residue in each frame of the MD trajectory using MdTraj's dictionary of protein secondary structure (DSSP 2.2.0) assignment and show the most frequently observed secondary structure label as color of each cell. The frequency to observe the most frequently observed label is annotated as percentage in each cell. Noticeably, Helix VIII (res.464-480) is solidly maintained as an α helix between G464 and A480.

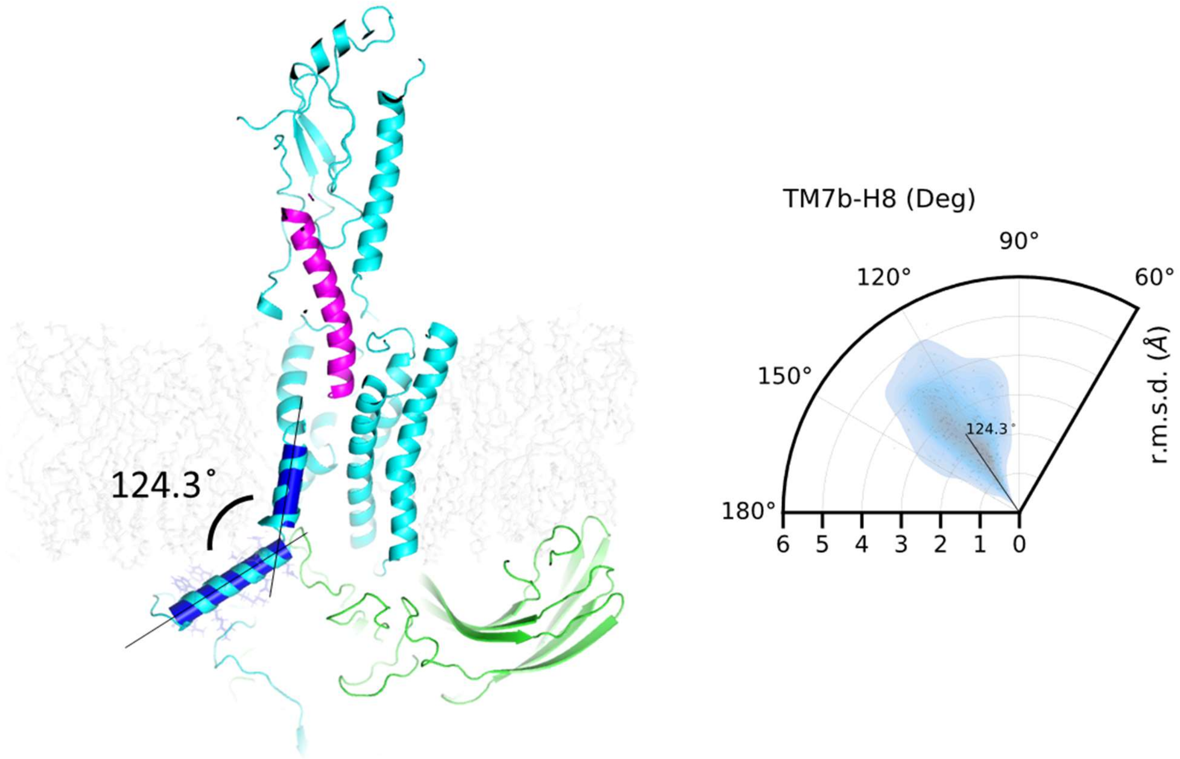

Supplementary Fig. 29: Orientation of Helix VIII in MD simulations. We measured the angle between the lower TM7b (res. 451-461) helix and the Helix VIII (res.464-480) and show the distribution of the angle together with the root mean squared distance (r.m.s.d.) of Helix VIII from its initial position. The angle between the two helices of concern is maintained at 124.3° in average with a standard deviation of 9.1° and r.m.s.d. at 2.4 angstrom in average with standard deviation of 1.0 Å.

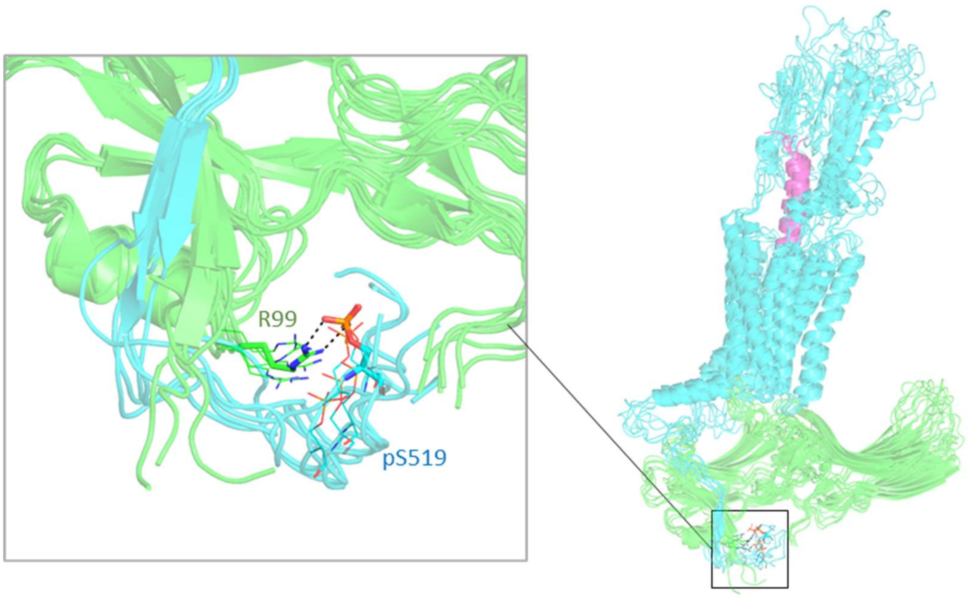

Supplementary Fig. 30: Single phosphorylation site pS519. Stacked snapshots of PTH1R pS519 interacting with R99. Snapshots were taken every 200 ns spanning 1200 ns.

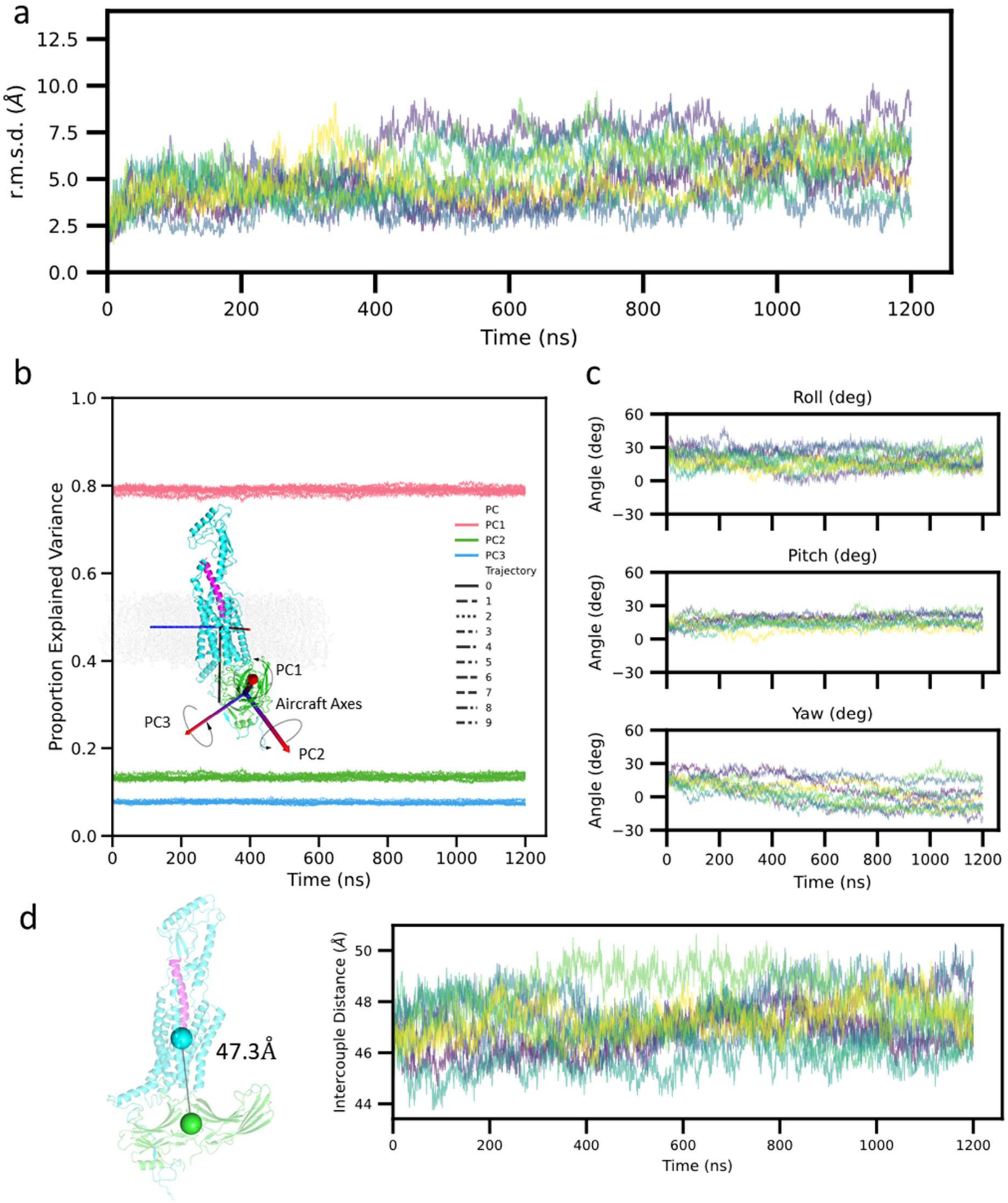

Supplementary Fig. 31: Orientation of arr2 in MD simulations. (a) Time series of r.m.s.d. from starting position of arr2. (b) Proportion of explained variance in principal axes (aircraft axes) of arr2 coordinate. In all trajectories, the principal axes are well distinguished by their explained variance throughout hence showing that the change in overall shape of arrestin (defined by its Cα coordinates) is minimal. This assures that the identity of principle axes is unambiguous and cannot interchange. (c) Time series of orientation angles. (d) Time series of intercouple distance, defined as the distance between mean of arrestin (green sphere) and mean of the 7TM core (res.182-460) (cyan sphere). The intercouple distance in our model is 47.3 Å

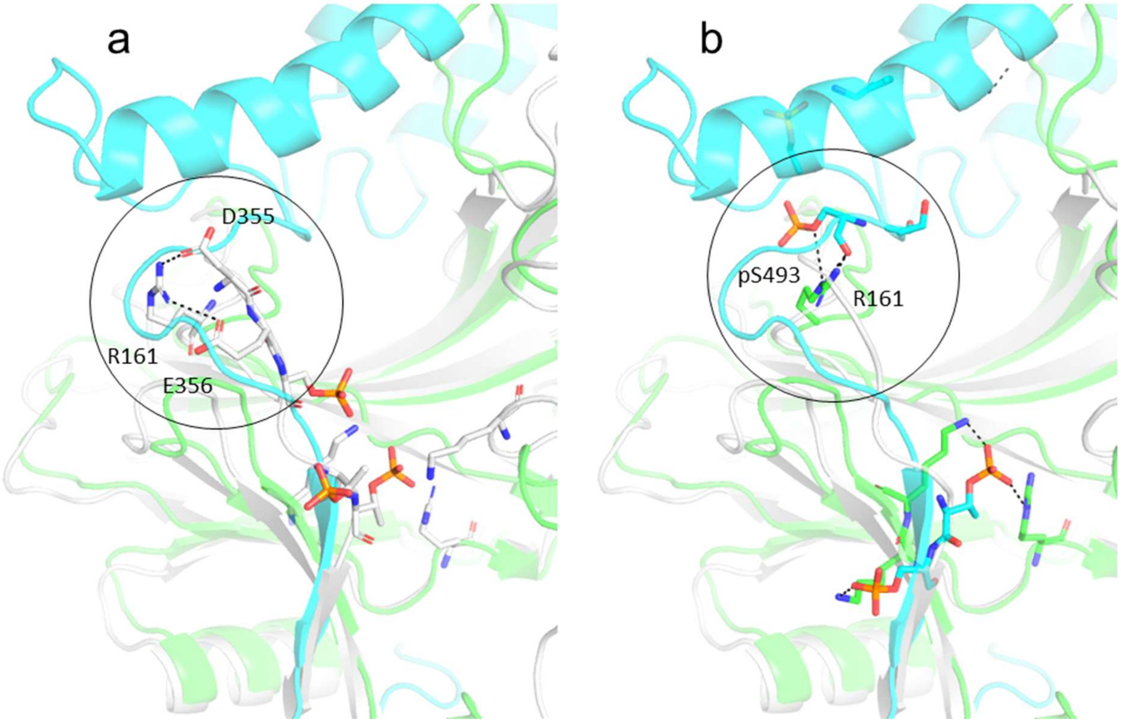

Supplementary Fig. 32: Ionic interactions between (a) aspartate/glutamate on cytoplasmic tail of β_2V_2R-Gs-arr2 megaplex (PDBID: 6ni2 corresponds to the arr2–V2Rpp portion shown in white, and PDBID: 6ni3 corresponds to β_2V_2R–Gs portion of the megaplex) and (b) proximal phosphorylation cluster of our PTH1R model (in cyan for PTH1R, green for arr2). Note that while local resolution does not allow for placement of side chains for D355 and E356 in the megaplex arrangement, cryo-EM density for this region was observed and it was suggested by the authors that both residues are in a position to interact with K160 and R161 on arr2.

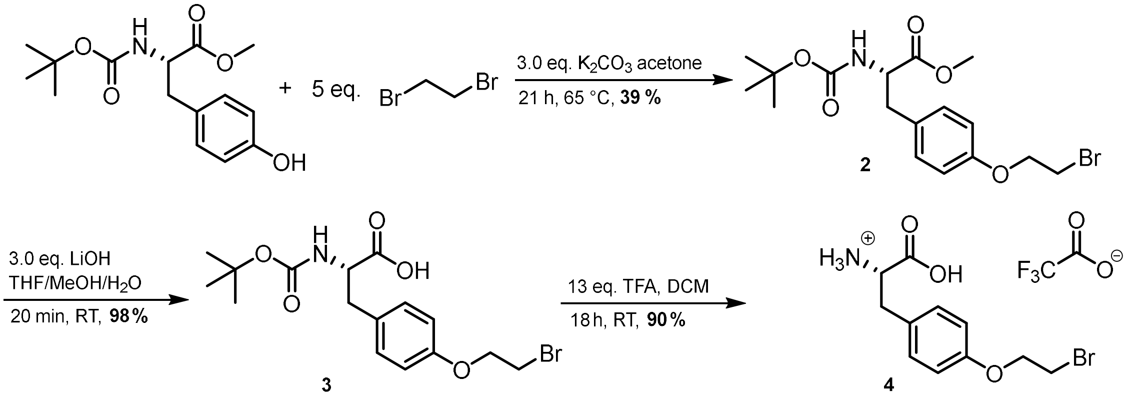

Supplementary Fig. 33: Reaction scheme for the synthesis of *O*-(2-bromoethyl)-L-tyrosine 4 (BrEtY)

SI Table

Supplementary Table 1: Literature survey of PTH1R phosphorylation. The following articles used a variety of experimental techniques to locate phosphorylation sites on the PTH1R. Positions not demonstrated by later in vitro mass spectrometry experiments are indicated with a δ, but these discrepancies could potentially arise due to differential accessibility of kinases in different cell lines and other experimental conditions. Phospho-site numbers refer to the human ortholog.

Author	Doi	Title	Phospho-Sites	Remark
**Blind et al 1996**	10.1002/jbmr.5650110505	Phosphorylation of the cytoplasmic tail of the PTH/PTHrP receptor	S473 δ<br>S475 δ<br>T478 δ<br>S491	To localize phosphorylation sites on the cytoplasmic tail, the authors prepared a recombinant fusion protein containing the rat PTH1R C-terminus to serve as substrate for phosphorylation by purified PKA, PKC, and GRK2 in vitro. They identified S491, together with minor sites at S473 and/or S475, as the preferential site for PKA-mediated phosphorylation. On the other hand, they showed that none of the three sites is a major target of PKC. For GRK2, little to no phosphorylation of the C-terminus fusion protein was found, likely due to the absence of the full-length receptor in the experiment, which activates GRK2.<br>By replacing serine/threonine with alanine in ICL3 of the opossum PTH1R expressed in HEK 293 cells, the authors concluded that these positions did not affect PTH-inducible phosphorylation nor basal phosphorylation in the cytoplasmic tail.
**Malecz et al 1998**	10.1210/mend.12.12.0203	Identification of Phosphorylation Sites in the G Protein-Coupled Receptor for Parathyroid Hormone. Receptor Phosphorylation Is Not Required for Agonist-Induced Internalization	S491<br>S492<br>S495 δ	In this study, the authors investigated phosphorylation sites with opossum PTH1R expressed in HEK 293 cells. By performing a series of phosphoamino acid analysis on ^{32}P-labeled PTH1R mutants, they identified S485, S486, or S489 (human: S491, S492 and S495) as sites of phosphorylation in presence of agonist. They also reported basal phosphorylation at S489. In addition, they also concluded that S467 and S469 (human: S473 and S475) were not phosphorylated, since no phosphothreonine appeared if threonine was substituted for either of the two serine residues. They also showed that overexpressed GRK2 enhanced phosphorylation of the wildtype PTH1R, but had no effect on the rate or extent of receptor internalization in response to PTH.
**Tawfeek et al 2002**	10.1210/mend.16.1.0760	Phosphorylation of the Receptor for PTH and PTHrP Is Required for Internalization and Regulates Receptor Signaling	S491<br>S492<br>S493<br>S495 δ<br>S501<br>S504	Using site-directed mutagenesis, phosphopeptide mapping, and direct sequencing of phospho-receptor fragments, the authors reported PTH-dependent phosphorylation of the rat PTH1R in COS-7 cells. Based on mutagenesis and radiosequencing data, they concluded that phosphorylation occurred on S491, S492, S493, S495, S501, and S504, but suggested that S489 was not phosphorylated due to insufficient radioactivity detected, though the serine in position 489 is required for phosphorylation. They also suggested that S491 is frequently phosphorylated and that either both of S492/S493 or only S493 is phosphorylated.
**Miedlich et al 2008**	10.1152/ajpendo.00036.2008	Eliminating phosphorylation sites of the parathyroid hormone	S489<br>S491<br>S492	This study explored the downstream effect of single and multiple mutagenesis on some phosphorylation sites of rat PTH1R in COS-7 cells. They concluded that preventing one or more phosphorylation of either S491/492/493, S501, or

		receptor type 1 differentially affects stimulation of phospholipase C and receptor internalization	S493 S495 δ S501 S504	S504 enhances coupling of the PTH1R to phospholipase C upon PTH stimulation. However, PTH-dependent inositol phosphate accumulation in cells with the phosphorylation-deficient mutant S489A/S491A/S492A/S493A/S495A/S501A/S504A, was much higher, suggesting that individual mutations do not fully recapitulate the phospho-deficient PTH1R phenotype.
**Zindel et al 2016**	10.1042/BCJ20160740	Identification of key phosphorylation sites in PTH1R that determine arrestin3 binding and fine-tune receptor signaling	S73 S473 S491 S492 S493 T503 S504 S519 T547 T551	In this study, LC–MS/MS sequencing was used to identify phosphorylation sites on tryptic phosphopeptides generated from immuno-purified human PTH1R with PTH stimulation in HEK293T cells. Ten sites were identified in total. Nine of these (S473, S491, S492, S493, T503, S504, S519, T547 and T551) were located in the C-terminus, while S73 is in the extra-cellular domain. Significantly higher abundance was reported for S491, S492/S493 and S519. Except for T503/S504, all other sites are singly phosphorylated. For S492/S493, either one of these is phosphorylated, the precise position of phosphorylation could not be determined. Phosphorylation on ICL3 were not detected in mass spectrometry, possibly due to lower coverage of this short loop. The authors also investigated interaction between PTH1R and arr3 using FRET and BRET experiments and concluded that the two P-clusters located at S489–S495 and at S501–T506 operate in concert to promote the formation of stable arrestin–receptor complexes. Significant FRET signal loss was observed with T503A/S504A mutantssuggesting that T503/S504 are the key determinant for high-affinity arrestin recruitment.
**Qiu et al 2018**	10.1038/s41413-017-0002-7	IGF-I induced phosphorylation of PTH receptor enhances osteoblast to osteocyte transition	Y494	By comparing mass spectra of phosphorylated and unphosphorylated peptides, the authors identified that Y494 on a recombinantly expressed human PTH1R C-terminus can be specifically phosphorylated by IGF1R tyrosine kinase upon induction by IGF1R in vitro. To track the phosphorylated PTH1R in osteoblasts and early osteocytes in vitro and in vivo, they developed a polyclonal antibody against the phosphorylation site.
**Zhang et al 2018**	10.1074/jbc.RA118.001737	Site-specific polyubiquitination differentially regulates parathyroid hormone receptor–initiated MAPK signaling and cell proliferation	T387 T392 S489 S491 S492 S493	In this study, reversible ubiquitination sites in human PTH1R was studied. Using tandem MS, the authors identified K388 in ICL3 and K484 in the proximal C-terminus as the primary ubiquitination sites in PTH1R. They also obtained MS/MS spectra to verify phosphorylation sites at ICL3 and the proximal C-terminus in PTH1R overexpressed in HEK-293S GnTI$^-$ cells. Several simultaneous phosphorylation were reported in the mass spectra, namely, T387/T392, S489, S491, S493, S491/S493, S491/S492, S489/S493. They also concluded that PTH1R binding to β-arrestin as well as ubiquitination are dependent on T387/T392 phosphorylation.
**White et al 2021**	10.1126/scisignal.abc5944	Spatial bias in cAMP generation determines biological responses to PTH type 1 receptor activation	T387 T392 S489 S491 S492 Y494 T503 S504 S519 T551	The authors identified ten phosphorylation sites in human PTH1R in response to PTH stimulation by SILAC-based LC-MS/MS. They noted that ICL3 phosphorylation status lacks quantitative information, potentially due to that ICL2/3 phosphorylation only takes part in initial β-arrestin recruitment. Except for S492/S504, all other sites were found to be singly phosphorylated.

Supplementary Table 2: Number of atoms in molecular dynamics simulation starting from M_2R-arr2-based model.

Content	Number of atoms
Water	167232
Sodium	152
Chloride	164
POPC	38592
Cholesterol	7104
Protein	14318
Total	227562
