# Supplementary material for: Structural details of a Class B GPCR-arrestin complex revealed by genetically encoded crosslinkers in living cells
Source: Nat Commun. 2023 Mar 1;14:1151. doi: 10.1038/s41467-023-36797-2 (PMC9977954; doi:10.1038/s41467-023-36797-2)
Supplement: Supplementary file 3 — Description of Additional Supplementary Files [file 41467_2023_36797_MOESM3_ESM.pdf]

File name: Supplementary Data 1

Description: Background signals in the PTH1R-arr2-complex. Relative intensity of the background signal running at the height of the crosslinking band (Supplementary Fig. 8) as a fraction of the total arrestin signal (crosslinked + not crosslinked) was determined by densitometric analysis of at least three biological replicates of the αHA western blots. P-value of the signal-to-noise ratio for each pair is given. Background signals of all positions with P-values < 0.02 (green highlight) were pooled and used as control group for the two-sided test (Welch's t-test) of each crosslinking pair (Supplementary Data 2).

File name: Supplementary Data 2

Description: Crosslinking pairs in the PTH1R-arr2-complex. Relative intensity of the crosslinking band as a fraction of the total arrestin signal (crosslinked + not crosslinked) as determined by densitometric analysis of at least three biological replicates of the α-HA western blots. Standard deviation among all replicates is given in percent. P-value of the signal-to-noise ratio for each pair as well as the p-value for the two-sided test (Welch's t-test) of each pair against the background signal control.

File name: Supplementary Data 3

Description: Plot of Distances against Crosslinking Yield in Fig 3a

File name: Supplementary Data 4

Description: Distribution of distances in Fig 5a

File name: Supplementary Data 5

Description: Distribution of distances in Fig 5b

File name: Supplementary Data 6

Description: Distribution of distances in Fig 5c

File name: Supplementary Data 7

Description: Orientation of Arrestin in Fig 5d, 5e and 5f
