# Supplementary material for: Structural details of a Class B GPCR-arrestin complex revealed by genetically encoded crosslinkers in living cells
Source: Nat Commun. 2023 Mar 1;14:1151. doi: 10.1038/s41467-023-36797-2 (PMC9977954; doi:10.1038/s41467-023-36797-2)
Supplement: Supplementary file 11 — Reporting Summary [file 41467_2023_36797_MOESM11_ESM.pdf]

Corresponding author(s): Coin, Irene

Last updated by author(s): Feb 6, 2023

Reporting Summary

Nature Portfolio wishes to improve the reproducibility of the work that we publish. This form provides structure for consistency and transparency in reporting. For further information on Nature Portfolio policies, see our Editorial Policies and the Editorial Policy Checklist.

Statistics

For all statistical analyses, confirm that the following items are present in the figure legend, table legend, main text, or Methods section.

n/a	Confirmed	
☐	☒	The exact sample size (*n*) for each experimental group/condition, given as a discrete number and unit of measurement
☐	☒	A statement on whether measurements were taken from distinct samples or whether the same sample was measured repeatedly
☐	☒	The statistical test(s) used AND whether they are one- or two-sided<br>*Only common tests should be described solely by name; describe more complex techniques in the Methods section.*
☒	☐	A description of all covariates tested
☐	☒	A description of any assumptions or corrections, such as tests of normality and adjustment for multiple comparisons
☐	☒	A full description of the statistical parameters including central tendency (e.g. means) or other basic estimates (e.g. regression coefficient) AND variation (e.g. standard deviation) or associated estimates of uncertainty (e.g. confidence intervals)
☐	☒	For null hypothesis testing, the test statistic (e.g. *F*, *t*, *r*) with confidence intervals, effect sizes, degrees of freedom and *P* value noted<br>*Give P values as exact values whenever suitable.*
☒	☐	For Bayesian analysis, information on the choice of priors and Markov chain Monte Carlo settings
☒	☐	For hierarchical and complex designs, identification of the appropriate level for tests and full reporting of outcomes
☒	☐	Estimates of effect sizes (e.g. Cohen's *d*, Pearson's *r*), indicating how they were calculated

Our web collection on statistics for biologists contains articles on many of the points above.

Software and code

Policy information about availability of computer code

Data collection	Mutagenesis: Primers were designed using AAscan software (GPLv3 licence, freeware). WB: immunostained blots were detected via ECL reaction and imaged in a G:BOX Chemi XL (Syngene); cAMP assay: Renilla luciferase luminescence signal was measured with an Omega plate reader (BMG LABTECH) equipped with a 475-30 nm emission filter; BRET recruitment assay: Nanoluc luciferase and Venus emission were measured with an Omega plate reader (BMG LABTECH) equipped with 475-30 nm and 520 nm emission filters.
Data analysis	WB: raw 16-bit tif images of anti-HA stained blots were imported in Image Studio Lite Ver 5.2 (Licor), arrestin and related crosslinking bands were measured via rectangular shapes in Analysis mode; cAMP and BRET recruitment assay: measurments were imported in Prism 9 (Graphpad) and curves were fitted by non-linear regression; Modeling was carried out with ICM-Pro v.3.9.2c (Molsoft LLC); MD simulation: CHARMM-GUI Version 3.6 (2021.July) webserver was used for input generation, membrane orientation was assigned by PPM 2.0 webserver via CHARMM-GUI, simulation was run using CHARMM36ff in Gromacs (v2020.1) on USC Center for Advanced Research Computing; GPC clusters analysis was facilitated by MDTraj package for Python. Secondary Structure Classification in MD simulations was run using DSSP 2.2.0.

For manuscripts utilizing custom algorithms or software that are central to the research but not yet described in published literature, software must be made available to editors and reviewers. We strongly encourage code deposition in a community repository (e.g. GitHub). See the Nature Portfolio guidelines for submitting code & software for further information.

Data

Policy information about availability of data

All manuscripts must include a data availability statement. This statement should provide the following information, where applicable:

- Accession codes, unique identifiers, or web links for publicly available datasets
- A description of any restrictions on data availability
- For clinical datasets or third party data, please ensure that the statement adheres to our policy

The authors declare that the data supporting the findings of this study are available within the paper and its supplementary information.
The coordinates of the best PTH1R-arr2 model, based on the M2R-arr2 template, have been deposited in the ModelArchive database under accession code ma-2b2xn [add hyperlink here], other models are deposited under ma-33nf3, ma-5ui3z, ma-9mi1q, ma-f1hkg, ma-v0m33.
The following protein structures were used in this paper: PDBID: 4jqi, PDBID: 6nbf, PDBID: 5w0p, PDBID: 6u1n, PDBID: 6tko, PDBID: 6pwc, 6up7, PDBID: 1g4m, PDBID: 7r0c, PDBID: 6ni2, PDBID: 6ni3. Source data are provided with this paper.

Human research participants

Policy information about studies involving human research participants and Sex and Gender in Research.

Reporting on sex and gender	N/A
Population characteristics	N/A
Recruitment	N/A
Ethics oversight	N/A

Note that full information on the approval of the study protocol must also be provided in the manuscript.

Field-specific reporting

Please select the one below that is the best fit for your research. If you are not sure, read the appropriate sections before making your selection.

☒ Life sciences ☐ Behavioural & social sciences ☐ Ecological, evolutionary & environmental sciences

For a reference copy of the document with all sections, see nature.com/documents/nr-reporting-summary-flat.pdf

Life sciences study design

All studies must disclose on these points even when the disclosure is negative.

Sample size	Western blots for the preliminary photo-crosslinking screening were done only once and repeated only in case of technical pitfalls (incomplete transfer to the membrane, antibody not working, etc.). The aim of this experiment is only to gain an overall idea of interaction regions. This information was not used for the construction of the models.<br>All chemical crosslinking experiments that were quantified have been performed at least in biological triplicates, i.e. distinct batches of cells were transfected in different days., thus yielding at least three independent western blots for quantification of signals.<br>Assays were performed at least in biological triplicates, each measured as technical quadruplicate, which is a standard sample size for this kind of experiments.<br>We carried out three to five repetitions of independent transient transfection, stimulation and downstream analysis of the WB samples. For assays, at least three independent experiments were carried out, each measured as technical quadruplicate.
Data exclusions	Replica experiment 4 for the background signal estimation was excluded due to subpar crosslinking yields judging from the positive control sample carried throughout all blots. Background signal experiment pair Arr2-156BrEtY+PTH1R-C397S (replica 5) was excluded due to handling error.
Replication	Experiments were repeated as described above and in the methods part or figure legends. All experiments shown could be reproduced as described.
Randomization	No experimental group randomization was carried out since experiments were carried out in live cells. Rendomization is not relevant nor would be applicable to this study.
Blinding	Experimenters were not blinded. This was not possible because cloning, DNA preparation, transfection and western blot were carried out by the same investigator.

Reporting for specific materials, systems and methods

We require information from authors about some types of materials, experimental systems and methods used in many studies. Here, indicate whether each material, system or method listed is relevant to your study. If you are not sure if a list item applies to your research, read the appropriate section before selecting a response.

Materials & experimental systems

n/a	Involved in the study
☐	☒ Antibodies
☐	☒ Eukaryotic cell lines
☒	☐ Palaeontology and archaeology
☒	☐ Animals and other organisms
☒	☐ Clinical data
☒	☐ Dual use research of concern

Methods

n/a	Involved in the study
☒	☐ ChIP-seq
☒	☐ Flow cytometry
☒	☐ MRI-based neuroimaging

Antibodies

Antibodies used	anti-HA (3F10), rat, 1:2,000 (Roche Disgnostics)<br>anti-PTH1R (4D2), mouse, 1:2,000 (Thermo Fisher Scientific)<br>anti-mouse-HRP, goat, 1:10,000 (Santa Cruz Biotechnology)<br>anti-rat-HRP, goat, 1:5,000 (Cell Signaling Technology)
Validation	https://www.sigmaaldrich.com/DE/en/product/roche/12158167001<br>https://www.thermofisher.com/antibody/product/PTH1R-Antibody-clone-4D2-Monoclonal/MA5-15676<br>https://www.scbt.com/p/goat-anti-mouse-igg-hrp<br>https://www.cellsignal.de/products/secondary-antibodies/anti-rat-igg-hrp-linked-antibody/7077

Eukaryotic cell lines

Policy information about cell lines and Sex and Gender in Research

Cell line source(s)	293T cells were from DSMZ ACC-635
Authentication	Cells were not further authenticated.
Mycoplasma contamination	Cells were regularly checked for mycoplasma contamination using MycoAlertTM Mycoplasma Detection Kit (Lonza).
Commonly misidentified lines<br>(See ICLAC register)	Not used.
